# Distinct TORC1 signalling branches regulate Adc17 proteasome assembly chaperone expression

**DOI:** 10.1242/jcs.261892

**Published:** 2024-07-22

**Authors:** Thomas D. Williams, Ifeoluwapo Joshua, Flavie Soubigou, Sylwia M. Dublanska, Rebecka Bergquist, Adrien Rousseau

**Affiliations:** ^1^ MRC-PPU, University of Dundee, Dundee DD1 5EH, UK; ^2^ Sir William Dunn School of Pathology, University of Oxford, Oxford OX1 3RE, UK

**Keywords:** Actin cytoskeleton, Mpk1 kinase, Slt2 kinase, Proteasome assembly chaperone, TORC1 signalling

## Abstract

When stressed, cells need to adapt their proteome to maintain protein homeostasis. This requires increased proteasome assembly. Increased proteasome assembly is dependent on increased production of proteasome assembly chaperones. In *Saccharomyces cerevisiae*, inhibition of the growth-promoting kinase complex TORC1 causes increased proteasome assembly chaperone translation, including that of Adc17. This is dependent upon activation of the mitogen-activated protein kinase (MAPK) Mpk1 and relocalisation of assembly chaperone mRNA to patches of dense actin. We show here that TORC1 inhibition alters cell wall properties to induce these changes by activating the cell wall integrity pathway through the Wsc1, Wsc3 and Wsc4 sensor proteins. We demonstrate that, in isolation, these signals are insufficient to drive protein expression. We identify that the TORC1-activated S6 kinase Sch9 must be inhibited as well. This work expands our knowledge on the signalling pathways that regulate proteasome assembly chaperone production.

## Teaser

**Summary:** TORC1-regulated proteasome assembly chaperone production is necessary for proteasome assembly and proteostasis. We identify Cell Wall Integrity and Sch9 signalling as mechanisms controlling this.

## INTRODUCTION

When cells are stressed, they need to rapidly adapt their proteome to maintain protein homeostasis and survive (Williams and Rousseau, 2022). Proteome adaptation requires both an increase in the degradative capacity of the cell, either through increased autophagy or proteasome assembly, and changes to the proteins being produced through altered transcription, translation or both. The environmental-sensing protein kinase complex TORC1 (mTORC1 in mammals) co-ordinates the processes of protein translation and degradation at a bulk level (Williams and Rousseau, 2022). When these interlocking aspects of protein homeostasis are perturbed, including during aging, diseases follow (Rousseau and Bertolotti, 2018).

**Fig. 1.**
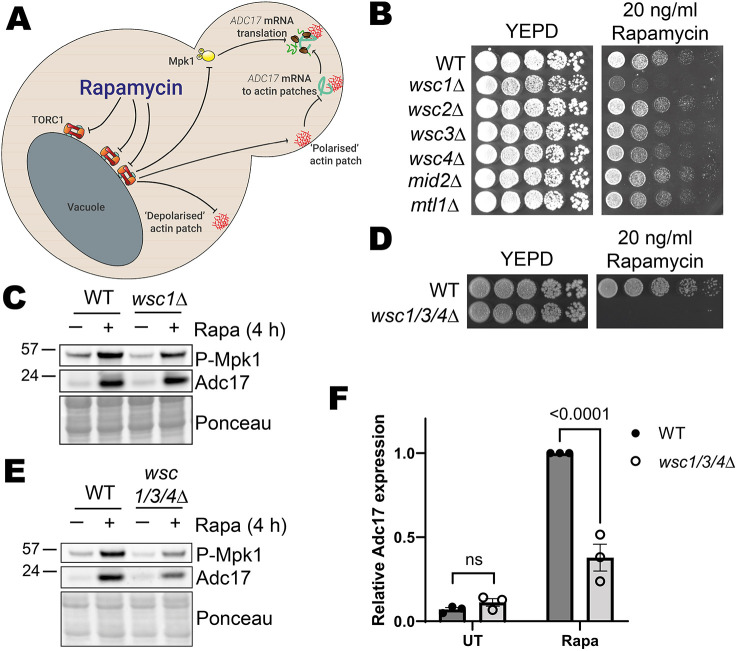
**Mpk1 is activated and actin depolarised via the Wsc1, Wsc3 and Wsc4 cell wall integrity pathway sensors in response to rapamycin treatment.** (A) Proteasome assembly chaperone translation regulation overview. Upon rapamycin treatment, TORC1 is inhibited, leading to activation of Mpk1 and *ADC17* mRNA relocalisation to cortical actin patches, together driving increased Adc17 production. (B) Drop assay for WT and CWI sensor mutants on YEPD and YEPD plus rapamycin after 3 days of growth. Representative of three experiments. (C) Mpk1 activation (P-Mpk1) and Adc17 levels following 4 h rapamycin treatment in WT and *wsc1Δ* cells. Representative of three experiments. (D) Drop assay of WT and *wsc1/3/4Δ* cells on YEPD and YEPD plus rapamycin after 3 days of growth. Representative of three experiments. (E) Mpk1 activation (P-Mpk1) and Adc17 levels following 4 h rapamycin treatment in WT and *wsc1/3/4Δ* cells. Representative of three experiments. (F) Quantification of the blots shown in E (mean±s.e.m., *n*=3, two-way ANOVA followed by a Tukey's multiple comparisons test). UT, untreated; Rapa, rapamycin treated.

Proteasome assembly induction upon nutrient stress is conserved from yeast to mammals (Gao et al., 2015; Rousseau and Bertolotti, 2016). The resulting increase in degradative capacity relies upon increased translation of proteasome assembly chaperones, including the yeast-specific stress-inducible chaperone Adc17, a model protein to explore increased proteasome assembly chaperone induction upon stress (Rousseau and Bertolotti, 2016; Williams et al., 2022, 2024). The induction of Adc17 expression at the level of translation is now fairly well described – translation of *ADC17* mRNA is reliant upon Mpk1 activation and mRNA relocalisation to actin patches following actin depolarisation (Fig. 1A) (Rousseau and Bertolotti, 2016; Williams et al., 2022, 2024).

Specifically inhibiting TORC1 via rapamycin treatment has long been known to cause actin depolarisation and activate Mpk1 by phosphorylation (Krause and Gray, 2002; Torres et al., 2002). Activated Mpk1 then acts to increase Mpk1 protein abundance, thus amplifying the cell wall integrity (CWI) signalling response (García et al., 2016). Both actin depolarisation and Mpk1 activation are well described as being downstream effects of CWI pathway activation (Jiménez-Gutiérrez et al., 2020). The CWI pathway was initially described as being activated in response to cell wall stress but has since been shown to be activated by many other stresses through the sensor proteins Wsc1, Wsc2, Wsc3, Wsc4, Mid2 and Mtl1. Wsc1 is the best characterised of these. Wsc1 has a cytosolic tail, a transmembrane domain, an extracellular ‘spring’ domain and a cysteine-rich head domain, which is required for clustering in distinct microdomains and possibly interaction with the cell wall (Dupres et al., 2009, 2011; Kock et al., 2016; Schöppner et al., 2022). Perturbations affecting the cell wall cause a conformational change in the cytosolic domain, allowing interaction with and activation of the RhoGEF Rom2 (Jendretzki et al., 2011). Rom2 activates Rho1, which triggers a kinase activation cascade from Pkc1 to Mpk1 via Bck1 and Mkk1 and Mkk2.

Although Mpk1 is activated downstream of TORC1 inhibition or CWI activation, it remains unclear whether TORC1 inhibition activates the CWI pathway or merely has a similar output. There have been conflicting reports on the potential role of the CWI sensor Mid2 in Mpk1 activation following TORC1 inhibition, and Wsc1 might also have a role (Krause and Gray, 2002; Torres et al., 2002). *wsc1Δ* and *mid2Δ* cells are not known to be rapamycin intolerant, as would be expected with impaired Mpk1 activation (Rousseau and Bertolotti, 2016). It therefore remains an open question as to whether Mpk1 is activated primarily through the CWI pathway following rapamycin treatment. To further complicate matters, actin depolarisation can activate the CWI pathway as well as this being triggered through CWI activation (Harrison et al., 2001). This raises the question of whether actin depolarisation induced by TORC1 inhibition is CWI dependent or independent, in addition to requiring Mpk1 activation.

Here, we clarify the links between TORC1, the CWI, actin architecture and Mpk1 activation in *Saccharomyces cerevisiae*. We show that TORC1 inhibition indeed activates the CWI to induce Mpk1 activation and actin rearrangements, but the S6 kinase Sch9 must also be inhibited for Adc17 protein expression. This work increases our knowledge of the signalling governing proteasome assembly chaperone production.

## RESULTS

### TORC1 regulates the CWI sensor proteins Wsc1, Wsc3, and Wsc4 to control Mpk1 activation

To obtain more detail about how the proteasome assembly pathway is induced following rapamycin treatment, we began by investigating the mechanisms of Mpk1 activation. Cells lacking Mpk1 are incapable of growth in the presence of rapamycin, a phenotype which is also observed in the upstream kinase deletion mutants *bck1Δ* and *mkk1Δ mkk2Δ* double mutants (Rousseau and Bertolotti, 2016). Therefore, strains that are incapable of Mpk1 activation following rapamycin treatment will likewise be incapable of growth in the presence of rapamycin. As Mpk1 is well described as being activated by a kinase cascade initiated by the CWI pathway, we focused on the most upstream element of this pathway – the CWI sensors.

We assessed the ability of knockouts for each of the six CWI sensor proteins to grow on rapamycin-containing plates (Fig. 1B). We included Wsc4 in this analysis, as although previous work using short-term induction has assigned its localisation to the ER, an endogenous knock-in we made showed a cortical localisation (Fig. S1A). All six mutants showed some level of growth, indicating functional redundancy between the sensor proteins. Three of the mutants, *wsc1Δ*, *wsc3Δ* and *wsc4Δ*, showed a consistent mild to moderate growth defect. The most severely affected strain, *wsc1Δ*, has previously been implicated in Mpk1 activation following rapamycin treatment (Krause and Gray, 2002). We did not, however, observe Mpk1 activation or a proteasome assembly chaperone (Adc17) induction defect using our *wsc1Δ* strain (Fig. 1C). Consistent with other studies, there was a basal level of Mpk1 activation present (Krause and Gray, 2002; Rousseau and Bertolotti, 2016; Torres et al., 2002; Williams et al., 2022).

We reasoned that there could be functional redundancy between Wsc1 and at least one other CWI protein. We therefore created double knockouts of Wsc1 with either the primary CWI protein Wsc2 or those whose mutants had a consistent growth defect, Wsc3 and Wsc4. The double mutant for Wsc1 and Wsc2 (*wsc1/2*Δ) had a similar level of residual growth on rapamycin as did *wsc1Δ* alone, whereas the double mutant strains for Wsc3 and Wsc4 (*wsc1/3*Δ) and Wsc1 and Wsc4 (*wsc1/4*Δ) failed to grow on rapamycin plates (Fig. S1B,C). Surprisingly, all of these double mutants were capable of robust Mpk1 activation and proteasome assembly chaperone induction upon rapamycin treatment (Fig. S1D,E). We therefore made a further mutant, removing all Wsc proteins which contributed to growth on rapamycin plates and found the resulting Wsc1, Wsc3 and Wsc4 triple mutant cells (*wsc1/3/4Δ*) were incapable of growth in the presence of rapamycin (Fig. 1D) and had greatly diminished Mpk1 activation and Adc17 proteasome assembly chaperone induction following rapamycin treatment (Fig. 1E,F). TORC1 inhibition therefore leads to activation of Mpk1 through the CWI pathway via the redundant activation of the CWI sensor proteins Wsc1, Wsc3 and Wsc4.

### CWI activation leads to actin depolarisation independently of Mpk1 activation

**Fig. 2.**
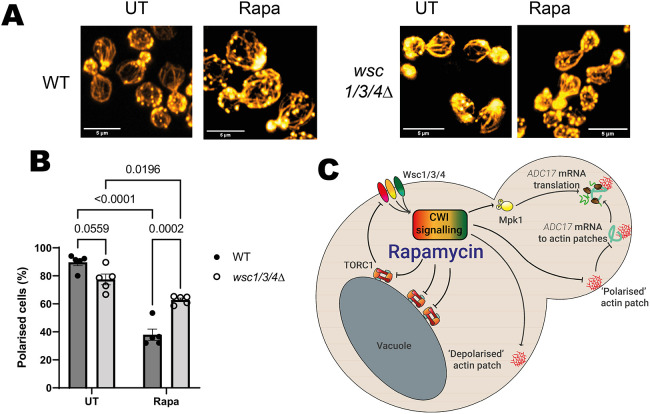
**Actin is depolarised via the CWI pathway sensors in response to rapamycin treatment.** (A) Effect of 1 h rapamycin treatment on actin polarisation in WT and *wsc1/3/4Δ* cells. Representative of five experiments. Cellular actin is labelled with rhodamine–phalloidin. Scale bars: 5 µm. (B) Quantification of images from A (mean±s.e.m., *n*=5, two-way ANOVA followed by a Tukey's multiple comparisons test). Budding cells are classified as unpolarised if there are six or more circular actin patches in the mother cell and polarised if this is not the case. (C) Updated scheme from Fig. 1A, showing role of Wsc1, Wsc3 and Wsc4 and CWI signalling in *ADC17* translation. UT, untreated; Rapa, rapamycin treated.

We next examined whether CWI activation caused by TORC1 inhibition leads to actin depolarisation, or whether the actin depolarisation leads to CWI activation. We analysed the effect of rapamycin on actin polarisation in *wsc1/3/4Δ* cells, where the CWI is impaired following rapamycin treatment (Fig. 1E). Following rapamycin treatment, WT cells become largely depolarised, whereas the *wsc1/3/4Δ* cells remain significantly more polarised (Fig. 2A,B). Actin depolarisation therefore occurs downstream of CWI activation following TORC1 inhibition.

We next tested whether actin depolarisation was regulated by Mpk1 activation. Deletion of Mpk1 did not inhibit actin depolarisation upon rapamycin treatment (Fig. S2A,B), unlike what has been previously observed in heat-stressed cells (Guo et al., 2009). Activating Mpk1 by inducible expression of a constitutively active version of the upstream kinase Mkk1, Mkk1^S377D;T381D^ (Mkk1-DD) (Harrison et al., 2004), likewise had no impact on actin polarity (Fig. S2C–E). CWI regulation of actin polarity downstream of TORC1 inhibition is therefore Mpk1independent. These data suggest that TORC1 inhibition activates the CWI through the Wsc1, Wsc3 and Wsc4 sensor proteins. CWI activation then causes both actin depolarisation and Mpk1 activation to regulate proteasome assembly chaperone translation (Fig. 2C).

### TORC1 inhibition activates the CWI by altering cell wall properties

It is unclear how inhibiting TORC1, a predominantly vacuole membrane-associated kinase complex, can activate Wsc1, Wsc3 and Wsc4 at the cell cortex. Although other reports suggest a potential role for the TORC1-regulated phosphatase Sit4, elevated Mpk1 activity in *sit4Δ* cells makes this unlikely (Torres et al., 2002). Linking the cell wall and TORC1, previous work has shown that TORC1 inhibition affects cellular resistance to zymolyase, a yeast cell wall-degrading enzyme (Krause and Gray, 2002). The observed increased zymolyase resistance, indicative of alterations in the cell wall, occurs after several hours of rapamycin treatment and is affected by the CWI-activated kinase Pkc1. When cells are provided with osmotic support (which can stabilise cells with weak cell walls), Mpk1 activation in response to rapamycin treatment is reduced (Torres et al., 2002). This suggests an effect at the cell wall might occur much earlier than previously described.

**Fig. 3.**
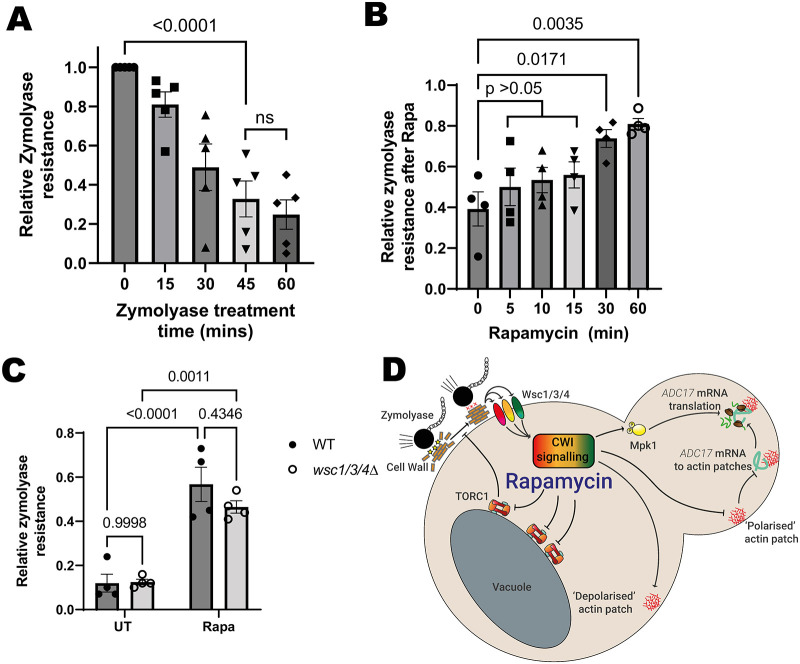
**Rapamycin treatment activates the CWL pathway by altering cell wall properties.** (A) Time courses of zymolyase resistance in WT cells growing exponentially in YEPD (mean with s.e.m., *n*=5, one-way ANOVA followed by a Tukey's multiple comparisons test). (B) WT cell resistance to 45 min zymolyase treatment after treatment with rapamycin (mean±s.e.m., *n*=4, one-way ANOVA followed by a Tukey's multiple comparisons test). (C) Resistance to 45 min zymolyase treatment by WT and *wsc1/3/4Δ* cells that were either untreated or after 1 h rapamycin treatment (mean±s.e.m., *n*=4, two-way ANOVA followed by a Tukey's multiple comparisons test). (D) Updated scheme from Fig. 1A, showing role of cell wall alterations, which increase zymolyase resistance in *ADC17* translation. UT, untreated; Rapa, rapamycin treated.

We followed the lysis of exponentially growing WT cells incubated with zymolyase by observing the reduction in their optical density at 600 nm (OD_600 nm_), which reached its lowest point after ∼45 min incubation (Fig. 3A). We used this 45 min timepoint for subsequent experiments. We assessed whether TORC1 inhibition affected cell wall properties by analysing zymolyase resistance in wild-type (WT) cells during the initial hour of rapamycin treatment (Fig. 3B). A trend of increasing zymolyase resistance was apparent from just 5 min of rapamycin treatment. This became statistically significant after 30–60 min. We examined whether this increased resistance was CWI dependent using the *wsc1/3/4Δ* strain, where CWI activation is impaired (Fig. 1E). WT and *wsc1/3/4Δ* cells that had not been treated by rapamycin had a comparably low resistance to zymolyase. For both strains, 60 min of rapamycin treatment caused a comparable increase in resistance to zymolyase (Fig. 3C). This indicates that there is no requirement for CWI activation for the changes responsible for increased zymolyase resistance. This is likely occurring through changes to the structure or composition of the cell wall. Increased resistance to zymolyase is therefore an early response to rapamycin treatment, before being later modulated by CWI activation.

TORC1 inhibition affects many downstream signalling branches, notably resulting in autophagy activation (Rabanal-Ruiz et al., 2017), S6 kinase inhibition (Urban et al., 2007) and PP2A phosphatase activation through Tap42 inhibition (Hughes Hallett et al., 2014). We used genetic manipulation to prevent these signalling changes from occurring (Fig. S3A). If a particular TORC1-regulated signalling branch was required for this change, it would be expected that a decrease in the zymolyase resistance would be observed after rapamycin treatment. Individually preventing activation of autophagy by *atg7* deletion, preventing phosphatase activity of Pph21, Pph22 or Sit4 through gene deletion, or inhibition of the major yeast S6 kinase Sch9 by introducing a constitutively active phospho-mimetic Sch9-2D3E (Sch9^T723D;S726D;T737E;S758E;S765E^) construct (Urban et al., 2007) had no effect on zymolyase resistance (Fig. S3B).

The largest change was observed when the Sch9-2D3E expression construct was used (Urban et al., 2007). Reasoning that there might be functional redundancy, we expressed this construct in the other strains and again tested zymolyase resistance in response to rapamycin treatment. When prevention of Sch9 inhibition was combined with prevention of activation of any of the phosphatases (Pph21, Pph22 or Sit4) we observed a statistically significant, albeit rather small, decrease in the resistance to zymolyase (Fig. S3C), which was not apparent when we used instead expressed WT Sch9 in these cells (Fig. S3D). Although it is established that Pph21 and Pph22 have similar, but not entirely overlapping, functions, the double mutant has either a substantial growth defect or is non-viable (Jonasson et al., 2016; Mitchell and Sprague, 2001; Shen et al., 2014). As reduced growth rates cause changes to the cell wall structure (McMurrough and Rose, 1967) and facilitates selection for suppressor mutants, we did not pursue whether deletion of both proteins might cause a stronger defect. In summary, TORC1 inhibition activates the CWI through altering cell wall properties to increase cellular zymolyase resistance (Fig. 3D). The nature of these changes, and the signalling processes involved, are an open question to be explored in later studies.

### CWI activation induces Adc17 production

**Fig. 4.**
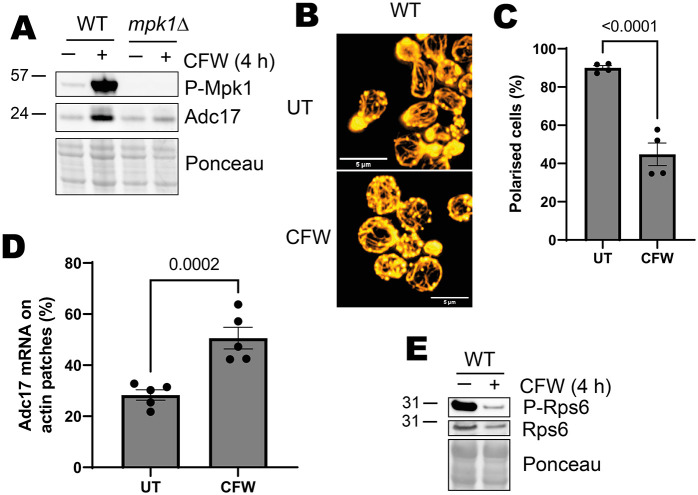
**Cell wall stress activates the CWI pathway, Adc17 protein expression and TORC1 inhibition.** (A) Mpk1 activation (P-Mpk1) and Adc17 levels in WT and *mpk1Δ* cells after 4 h Calcofluor White treatment. Representative of three experiments. (B) Effect of 1 h Calcofluor White treatment on actin polarisation in WT cells. Representative of four experiments. Cellular actin is labelled with rhodamine–phalloidin. Scale bars: 5 µm. (C) Quantification of images from B (mean±s.e.m., *n*=4, unpaired two-tailed *t*-test). Budding cells are classified as unpolarised if there are six or more circular actin patches in the mother cell and polarised if this is not the case. (D) Quantification of *ADC17* mRNA localised to actin patches in untreated or 1 h Calcofluor White-treated WT cells (mean±s.e.m., *n*=5, unpaired two-tailed *t*-test). (E) TORC1 activity (P-Rps6) and Rps6 levels in WT cells after 4 h Calcofluor White treatment. Representative of three experiments. UT, untreated; CFW, Calcofluor White treated.

To gain further detail on proteasome assembly pathway regulation, we examined whether rapamycin-induced CWI activation was sufficient for Adc17 production. We therefore bypassed TORC1, activating the CWI pathway directly using the chitin-binding compound Calcofluor White (CFW), which is well described as a CWI activator (Elorza et al., 1983). WT cells treated with CFW showed strong CWI induction [assessed by determining the levels of activated phosphorylated (P-)Mpk1] and Adc17 production (Fig. 4A). Adc17 protein expression was dependent on Mpk1 activation, as for rapamycin-treated cells (Fig. 4A) (Rousseau and Bertolotti, 2016). Activating the CWI pathway using micafungin, an echinocandin that inhibits β-1,3-glucan production, similarly induced Mpk1 activation and Adc17 protein production (Fig. S4A). We also observed the expected actin depolarisation and *ADC17* mRNA relocalisation to actin patches upon CFW treatment (Fig. 4B–D). However, the levels of TORC1-dependent phosphorylated Rps6 (P-Rps6) were reduced following CFW treatment (Fig. 4E), possibly through the action of Rho1 (Yan et al., 2012). This could not be explained by a reduction in Rps6 protein. We could therefore not rule out the involvement of other TORC1-regulated signalling in Adc17 protein expression following CWI pathway induction.

### Mpk1 activation and *ADC17* mRNA association with actin patches are necessary, but not sufficient, for Adc17 protein expression

**Fig. 5.**
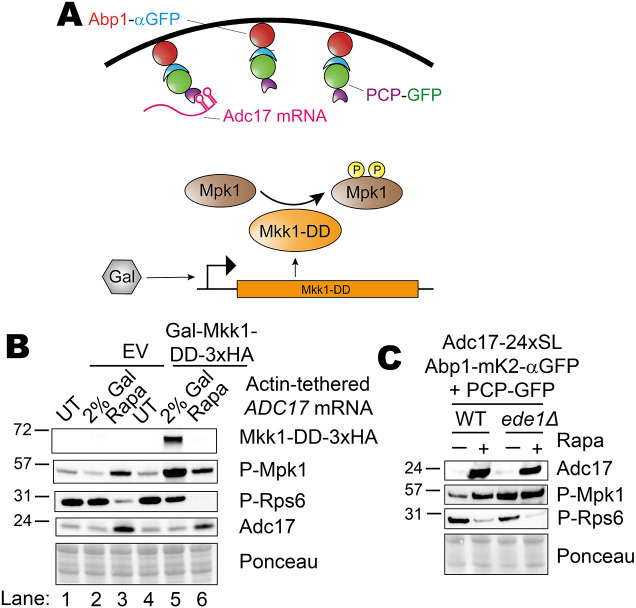
**Mpk1 activation and ADC17 mRNA recruitment to CAPs is insufficient for Adc17 translation induction.** (A) Scheme showing Mpk1 activation through inducible expression of the Mpk1 upstream kinase Mkk1 with the constitutively active ‘DD’ mutations (bottom) and *ADC17* mRNA tethering to actin patches using the actin patch protein Abp1 tagged with an anti-GFP nanobody (top), which binds GFP-tagged PP7 coat protein (PCP), which in turn binds *ADC17* mRNA with PP7 stem loops inserted into the 3′ UTR. (B) Mkk1-DD-3xHA and Adc17 levels, and Mpk1 and TORC1 activation (P-Mpk1 and P-Rps6, respectively) following 4 h rapamycin treatment or galactose induction in the cells with actin patch-tethered ADC17 mRNA (Adc17-24xSL Abp1-αGFP+PCP-GFP as described in Fig. 3A). Representative of at least 3 experiments. (C) Adc17 expression and Mpk1 and TORC1 activity (P-Mpk1 and P-Rps6, respectively) untreated and following 4 h rapamycin treatment in WT and *ede1Δ* cells with *ADC17* mRNA tethered to actin patches. Representative of at least 3 experiments.

As CWI activation leads to TORC1 inhibition, we looked downstream of CWI activation to assess whether *ADC17* mRNA relocalisation to cortical actin patches (CAPs) and Mpk1 activation were sufficient of for Adc17 protein expression. We tethered *ADC17* mRNA containing PP7 stem loops in the 3′ untranslated region (UTR) to actin patches by expressing the PP7-binding protein, PCP, fused to GFP in cells containing a nanobody against GFP fused to the CAP protein Abp1 (Fig. 5A, top), which we have previously shown can support a high level of Adc17 protein production (Williams et al., 2022). To these cells we added our inducible Mpk1 activation system, which is induced by galactose supplementation (Fig. S1D; Fig. 5A, bottom) (Williams et al., 2022). Combining these two features was insufficient to elevate Adc17 protein levels in the absence of rapamycin (Fig. 5B). Cells containing empty vector alone had no change in Adc17, P-Mpk1, P-Rps6 (TORC1 activation) or Mkk1-DD protein expression after 4 h of galactose supplementation (Fig. 5B, compare lanes 1 and 2), whereas rapamycin treatment had the expected effect (Fig. 5B, lane 3). Galactose supplementation therefore does not activate the CWI pathway and proteasome assembly chaperone production. Cells containing the vector saw increased levels of Mkk1-DD and, as expected, P-Mpk1 (Fig. 5B, compare lanes 4 and 5), to an even greater extent than when they were treated with rapamycin (Fig. 5B, lane 6). The effects on TORC1 activity (P-Rps6) were far less dramatic, however, and Adc17 levels were not increased, unlike when cells were treated with rapamycin (Fig. 5B, compare lanes 5 and 6). In conclusion, Mpk1 activation and *ADC17* mRNA localisation to CAPs is not sufficient for enhanced Adc17 protein production, likely due to TORC1 remaining active.

As our Mpk1 activation system requires the absence of glucose, we examined whether this was a factor by using *ede1Δ* cells, which have elevated Mpk1 activity, with actin-patch tethered *ADC17* mRNA, as these cells have elevated Mpk1 activation (Fig. 5C). These cells are likewise incapable of increased Adc17 protein production in the absence of rapamycin. An additional factor of factors downstream of TORC1 inhibition must therefore also be involved.

### Sch9 inhibition is important for Adc17 translation

**Fig. 6.**
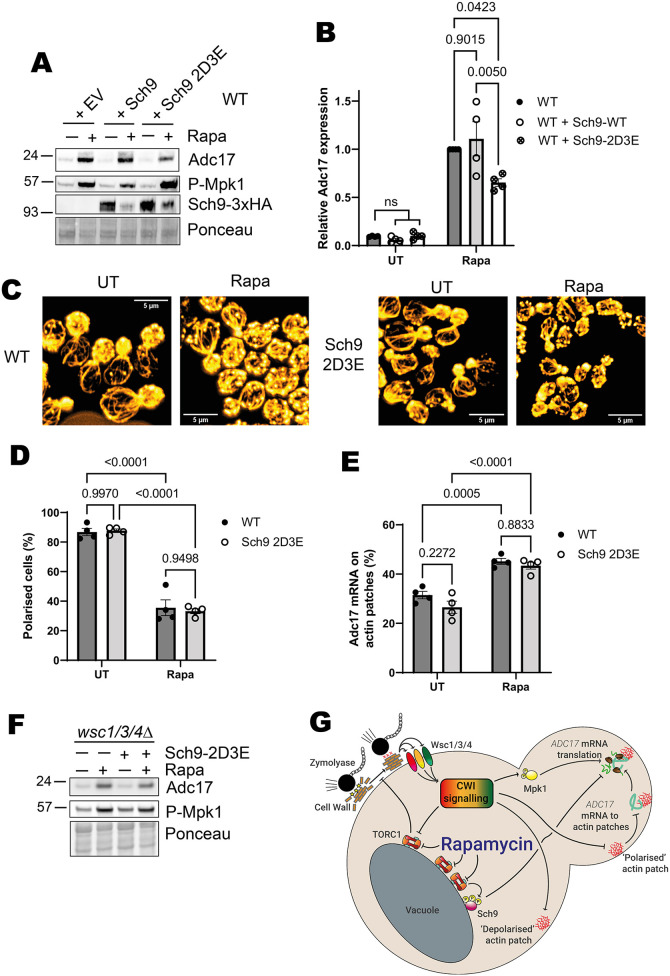
**Sch9 inhibition is required for rapamycin-induced Adc17 expression by a CWI-independent mechanism.** (A) Mpk1 activation (P-Mpk1) and Adc17 levels following 4 h rapamycin treatment in WT cells containing either empty vector (EV), WT Sch9 or the Sch9-2D3E constitutively active mutant. Representative of four experiments. (B) Quantification of blots shown in A (mean±s.e.m., *n*=4, two-way ANOVA followed by a Tukey's multiple comparisons test). (C) Effect of 1 h rapamycin treatment on actin polarisation in WT and Sch9-2D3E expressing cells. Representative of four experiments. Cellular actin is labelled with rhodamine-phalloidin. Scale bars: 5 µm. (D) Quantification of cellular actin polarisation from images shown in C (mean±s.e.m., *n*=4, two-way ANOVA followed by a Tukey's multiple comparisons test). Budding cells are classified as unpolarised if there are 6 or more circular actin patches in the mother cell and polarised if not. (E) Quantification of *ADC17* mRNAs localised to actin patches in WT and Sch9-2D3E expressing cells from C (mean±s.e.m., *n*=4, two-way ANOVA followed by a Tukey's multiple comparisons test). (F) Effect of introducing constitutively active Sch9-2D3E into *wsc1/3/4Δ* cells on the response to rapamycin. Representative of three experiments. (G) Updated scheme from Fig. 1A, showing additional role of Sch9 in Adc17 protein translation. UT, untreated; Rapa, rapamycin treated.

To assess the involvement of factors downstream of TORC1, we returned to our mutant collection where signalling from individual TORC1 branches is maintained following TORC1 inhibition (Fig. S3A). We assessed Adc17 production in these mutants following rapamycin treatment, finding no change in autophagy-deficient and Pph21 or Pph22 deletion mutants, whereas Sit4 deletion had a small effect on Adc17 production (Fig. S5A–C). Excitingly, there was a notable decrease in Adc17 induction in cells expressing the constitutively active Sch9-2D3E, suggesting a role for Sch9 inhibition (Fig. 6A,B). This was not due to compromised Mpk1 activation (Fig. 6A). Notably, *sch9Δ* cells also had a defect in Adc17 production, but not Mpk1 activation (Fig. S5D,E), demonstrating the importance of Sch9 activity modulation for proteasome assembly chaperone production.

We assessed whether preventing Sch9 inhibition could modulate *ADC17* mRNA localisation. Sch9-2D3E expression did not compromise actin depolarisation (Fig. 6C,D) or *ADC17* mRNA relocalisation to actin patches (Fig. 6E). The number of mRNA puncta was similar between the WT and Sch9-2D3E cells, indicating that the decrease in Adc17 protein production is unlikely to be due to a change in *ADC17* mRNA abundance (Fig. S5F). TORC1 inhibition therefore promotes Adc17 protein expression through both CWI pathway activation and Sch9 inactivation.

We assessed whether the reduction in Adc17 protein induction following rapamycin we had observed in the *wsc1/3/4Δ* mutant (Fig. 1E,F) could be enhanced by expressing constitutively active Sch9-2D3E in these cells (Fig. 6F). Maintaining Sch9 activation had no effect on Mpk1 activation in the *wsc1/3/4Δ* cells or Adc17 protein induction. CWI pathway activation through Wsc1, Wsc3 and Wsc4 is therefore the major regulatory pathway in *ADC17* mRNA translation, whereas Sch9 inhibition has an important role in modulating the downstream response (Fig. 6G).

### ADC17 mRNA localisation to CAPs is dispensable for chronic Adc17 protein expression

**Fig. 7.**
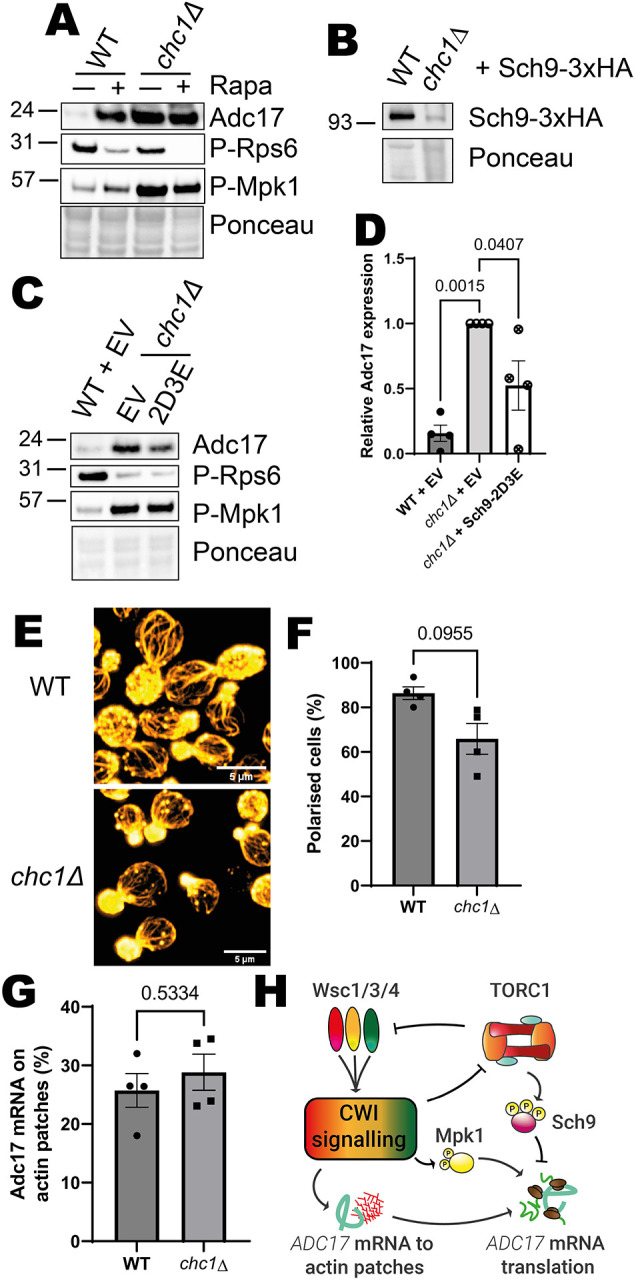
**Absence of Chc1 chronically activates the proteasome assembly pathway through Mpk1 activation and Sch9 inhibition.** (A) Mpk1 activation (P-Mpk1) and Adc17 levels following rapamycin treatment in WT and *chc1Δ* cells. Representative of four experiments. (B) Sch9 levels in WT and *chc1Δ* cells. Representative of three experiments. (C) Mpk1 activity (P-Mpk1), TORC1 activity (P-Rps6) and Adc17 levels in WT and *chc1Δ* cells containing either empty vector (EV) or expressing Sch9-2D3E. Representative of four experiments. (D) Quantification of Adc17 levels from C (mean±s.e.m., *n*=4, one-way ANOVA followed by a Tukey's multiple comparisons test). (E) Actin polarisation in WT and *chc1Δ* cells. Representative of four experiments. Cellular actin is labelled with rhodamine-phalloidin. Scale bars: 5 µm. (F) Quantification of actin polarisation from images shown in D (mean±s.e.m., *n*=4, unpaired two-tailed *t*-test). Budding cells are classified as unpolarised if there are 6 or more circular actin patches in the mother cell and polarised if not. (G) Quantification of *ADC17* mRNAs localised to actin patches in untreated WT and *chc1Δ* cells (mean±s.e.m., *n*=4, unpaired two-tailed *t*-test). (H) Scheme showing the proposed signalling control of Adc17 expression.

In a previous screen, we identified high levels of basal Adc17 in cells lacking the clathrin heavy chain Chc1 (Williams et al., 2022). We examined whether the aspects of the proteasome assembly pathway we have thus far uncovered were active in *chc1Δ* cells. Under basal conditions, *chc1Δ* cells not only have elevated Adc17, comparable to WT cells treated with rapamycin, they also have extremely high levels of active Mpk1 and low levels of TORC1 activity, as visualised by P-Rps6 (Fig. 7A), consistent with our model (Fig. 6G).

We observed very low levels of Sch9 in *chc1Δ* cells, confirming that its overall function is decreased in *chc1Δ* cells compared to WT cells (Fig. 7B). This could be due to the reduction in TORC1 activity, as rapamycin treatment also caused reduced levels of Sch9 (Fig. 6A). Increasing Sch9 activity by expressing the Sch9-2D3E phospho-mimetic protein reduced Adc17 levels without affecting the activation status of either TORC1 or Mpk1 (P-Rps6 and P-Mpk1, Fig. 7C,D).

Surprisingly, given the low levels of TORC1 activity, *chc1Δ* cells largely maintain actin polarity (Fig. 7E,F) (Henry et al., 2002). Consistent with this, a similar proportion of *ADC17* mRNA localised to actin patches in WT and *chc1Δ* cells (Fig. 7G). Although *ADC17* mRNA relocalisation to actin patches might provide a boost to translation following TORC1 inhibition, it is not necessary to sustain high levels of the protein when cells have chronic TORC1 inhibition and Mpk1 activation. It is important to note here that although there was not an increase in *ADC17* mRNA localised to actin patches, a significant proportion (∼28%) was still present in this location. This level of localisation is likely to be sufficient to sustain the high level of protein when Mpk1 is active and Sch9 is inhibited.

Altogether, this work demonstrates that the proteasome assembly pathway is regulated by both Mpk1 and Sch9 kinases downstream of TORC1 inhibition and CWI activation. CWI regulation of *ADC17* mRNA relocalisation to actin patches can provide a translational boost but is not required under chronic TORC1 inhibition and CWI activation (Fig. 4H).

## DISCUSSION

Cells encounter changing environments multiple times a day, requiring them to alter their proteomes both by increasing translation and modulating protein degradation (Williams and Rousseau, 2024). A key element in this response, conserved through eukaryotes, is the TORC1 kinase complex. The activity of TORC1 is modulated in response to almost all stresses, making it central to cellular adaptation to environmental change. Although TORC1 inhibition can acutely increase proteasome abundance to aid proteome adaptation, the effectors involved, and how they are regulated, were previously unclear. In this work we identified two key effector branches: the cell wall integrity (CWI) pathway governing both actin polarity (and hence mRNA localisation) and Mpk1 MAPK activation, and the translational regulator Sch9.

The CWI pathway is known to be activated in response to multiple stresses, including many with no clear link to the cell wall, such as ER stress (Jiménez-Gutiérrez et al., 2020). It is possible that at least some stresses might cause TORC1 inhibition and thus activate the CWI pathway through this means. Supporting this theory, ER stress induces a similar increase of proteasome assembly to that seen upon direct TORC1 inhibition, requiring activation of the CWI-regulated kinase Mpk1 (Rousseau and Bertolotti, 2016).

Regulation of stress-adaptive protein levels by multiple TORC1 effector branches has been previously reported. For example, in mammalian cells, Nanog291 and Snail85 translation is stimulated by the combination of mTORC1 (the mammalian equivalent of TORC1) inhibition, and integrated stress response activation (Jewer et al., 2020). By using several layers of regulation, the cell ensures it does not mount an energetically expensive response when not required. This is important, as previous work has shown that both CWI pathway activation and Sch9 inhibition can be induced independently of TORC1, allowing either element to override the other. The CWI pathway is activated by stresses directly targeting the cell envelope, such as the Calcofluor White and micafungin used in this work. The intensity of the stress likely dictates which stress response branches are activated, thereby matching the response to the stress experienced. Sch9 can be activated by TORC1 only when localised to the same membrane – dissociation from the vacuolar membrane is crucial for complete Sch9 signalling inhibition upon stress (Novarina et al., 2021; Takeda et al., 2018). These checkpoints allow tuning of the extent of stress-induced proteasome assembly to the level of stress experienced.

Although we have identified several key elements in stress-induced production of the model proteasome assembly chaperone Adc17, others remain to be determined. The factors affecting increased *ADC17* mRNA transcription upon TORC1 inhibition are unknown, save that they appear to be unrelated to either Mpk1 or Sch9. It is also unclear how either Mpk1 activation or Sch9 inhibition increase Adc17 protein expression. S6 kinases like Sch9 have been shown to promote general translation through promoting ribosome biogenesis and promoting both ribosome scanning to the initiation codon and elongation (Williams and Rousseau, 2024). Thus, it is possible that by altering these translational processes Sch9 inhibition can change the types of mRNAs being translated. Sch9 has also been genetically linked with Rho5, with a double deletion being synthetically lethal, a phenotype suggested to occur through the subsequent uncontrolled activation of the common target Rim15. Rho5 is a small GTPase that becomes GTP bound upon stress and acts to limit CWI activation (Novarina et al., 2021; Schmitz et al., 2002, 2015). It is possible that constitutive Sch9 activation interferes with GTP binding of Rho5, either affecting the role of Rho5 in restricting CWI activation or another role important for Adc17 translation. The exact role of Sch9 will be explored in future work.

The described regulatory network for *ADC17* mRNA translation has the exciting potential to be applied to other mRNAs whose translation is induced upon stress. Mpk1 activation, actin depolarisation and Sch9 inhibition are each commonly observed upon stress in *S. cerevisiae*. How widely this signalling control can be applied across different stresses will be an exciting area for future study.

## MATERIALS AND METHODS

### Yeast strains and growth and treatment conditions

All strains used were derived from the BY4741 collection and are listed in Table S1. Mutants were made using homologous recombination as described previously (Agrotis et al., 2023; Black et al., 2023; Williams et al., 2022). Cells were grown at 30°C on YPD agar plates or in YEPD medium (YPD plates, 10 g/l yeast extract, 20 g/l peptone, 2% glucose, 20 g/l agar; YEPD medium, 10 g/l yeast extract, 20 g/l peptone, 2% glucose), with shaking at 200 rpm. For all experiments, cells were grown overnight prior to being diluted to 0.2 OD_600 nm_, grown to 0.4–0.6 OD_600 nm_ and used as described below.

For drop assays, cells were adjusted to 0.2 OD_600 nm_ in YEPD medium, and serially diluted 1/5 (such that each spot was with one fifth the cells of the previous spot) before being spotted on YEPD plates with or without 20 ng/ml rapamycin (LC Laboratories, R5000). Plates were then imaged after 3 days.

For western blot experiments, cell concentration was adjusted to 0.2 OD_600 nm_, and cells were grown until they reached ∼0.4–0.6 OD_600 nm_. They were then diluted back to 0.2 OD_600 nm_, split into 4 ml (untreated) and 12 ml [treated with 200 ng/ml rapamycin, 0.5 mg/ml Calcofluor White (Sigma-Aldrich 910090) or 5 µM micafungin (Cayman Chemical 18009)] and returned to the incubator for 4 h. Cells were then pelleted (3200 ***g*** for 4 min), flash-frozen in dry ice, and stored at −20°C overnight prior to extraction.

For galactose-inducible expression of Mkk1-DD from the Gal10 promoter to activate Mpk1, cells were grown to logarithmic phase in YEP plus 2% raffinose (10 g/l yeast extract, 20 g/l peptone and 2% raffinose), before galactose was added to 2% to initiate expression.

For microscopy experiments, cells were split into 2×4 ml cultures and either fixed straight away with 3.7% formaldehyde (final concentration) or subjected to treatment (200 ng/ml rapamycin or 0.5 mg/ml Calcofluor White) for 1 h, then fixed. Cells were incubated with the formaldehyde mixture for 20 min shaking at 30°C, then spun down (3200 ***g*** for 4 min) and washed twice in PBS before actin was labelled using phalloidin and cells were mounted onto slides as described previously (Williams et al., 2022).

### Protein extraction and western blotting

Cell pellets were washed once in 600 ml ice-cold water, once in 400 µl ice-cold 2 M lithium acetate, and once in 400 µl ice-cold 0.4 M NaOH before resuspension in 110 µl lysis buffer containing Roche cOmplete protease inhibitor cocktail (04693116001) and phosphatase inhibitors (Roche PhosSTOP and PHOSS-RO) (used at a concentration of 1 tablet per 10 ml lysis buffer). These were then placed in a heat block at 90°C for 10 min before addition of 2.64 µl 4 M acetic acid, and then returned to the heat block for 10 min. The supernatants were cleared by centrifugation (20,000 ***g*** for 10 s), and the protein concentration determined by measuring the sample absorbance at 280 nm using the standard nanodrop (Thermo Fisher Scientific) program, which was then normalised between samples.

Samples were diluted in 5× loading buffer (0.25 M Tris-HCl pH 6.8, 50% glycerol, 0.05% Bromophenol Blue) and ∼30 µg were run on a 6–14% SDS-PAGE gel as described before (Williams et al., 2022) for semi-dry western blotting (Bio-Rad TurboID). Ponceau-stained images were taken, membranes cut up and blocked with 5% milk for at least 1 h, and primary antibodies diluted in TBS plus 0.1% Tween 20 (TBS-T plus 5% BSA) and 0.02% sodium acetate were added (anti-P-Mpk1, rabbit, 1:1000, Cell Signaling Technology, 4370S; anti-Adc17, sheep, 1:200, Division of Signal Transduction Therapy, University of Dundee, UK, DU66321; anti-P-Rps6, rabbit, 1:1000, Cell Signaling Technology, 2211; anti-HA, mouse, 1:1000, Santa Cruz Biotechnology, sc-7392; anti-Rps6, rabbit, 1:1000, Cell Signalling Technology, 2217S) overnight. In the morning, membranes were washed and incubated with appropriate secondary antibody diluted in TBS-T (HRP-conjugated anti-mouse-IgG, 1:10,000, Cell Signaling Technology, 7076S; HRP-conjugated anti-Rabbit-IgG, 1:10,000, Cell Signaling Technology, 7074S; or HRP-conjugated anti-Sheep-IgG, 1:5000, Sigma-Aldrich, A3415) for at least an hour before being washed and revealed (BioRad Clarity or Clarity Max ECL). All western blot imaging was performed using a BioRad Chemidoc system. Uncropped blots are available in Fig. S6.

Quantifications were performed in FIJI using the measure tool to obtain band intensities. To account for loading differences, the intensity of Ponceau stain in each lane was taken and normalised to an average of 1. The band intensity was then divided by the normalised Ponceau amount and normalised to a control lane, usually WT treated with rapamycin.

### Microscopy and analysis

Wsc4–GFP cells were made by homologous recombination as previously described (Williams et al., 2022). For live-cell microscopy of Wsc4–GFP cells, the cells were grown overnight in SC medium, then diluted into fresh medium at an OD_600 nm_ of 0.2. They were allowed to grow for 4 h to reach log-phase before being imaged on a Zeiss 880 using the Airyscan mode with a 63× lens.

For fixed samples, following sample preparation, cells were imaged on a Zeiss 880 using the Airyscan mode and a 63× lens to take *Z*-stacks covering the entire cell layer. These were subject to a *Z*-projection in FIJI, and actin polarisation and mRNA localisation were scored in FIJI as previously described (Williams et al., 2022).

### Zymolyase assays

We adapted and simplified a previously described protocol for our assay (Kuranda et al., 2006). 1 OD_600 nm_ of log-phase cells, either treated with rapamycin for the indicated time or not, were spun down at 7200 ***g*** for 3 min, and washed twice in 5 ml water. Cells were then resuspended in 1 ml 0.1 M Tris-HCl pH 7.4, and the OD_600 nm_ measured before and after 45 min incubation, unless otherwise indicated, at 37°C with 1.3 units zymolyase (Stratech, MP Bioscience).

### Statistical analysis

Statistical tests were performed as stated in the figure legends using Graphpad PRISM.

## Supplementary Material



10.1242/joces.261892_sup1 Supplementary information
