# Supplementary material for: Distinct TORC1 signalling branches regulate Adc17 proteasome assembly chaperone expression
Source: J Cell Sci. 2024 Jul 22;137(14):jcs261892. doi: 10.1242/jcs.261892 (PMC11298713; doi:10.1242/jcs.261892)
Supplement: Supplementary information [file joces-137-261892-s1.pdf]

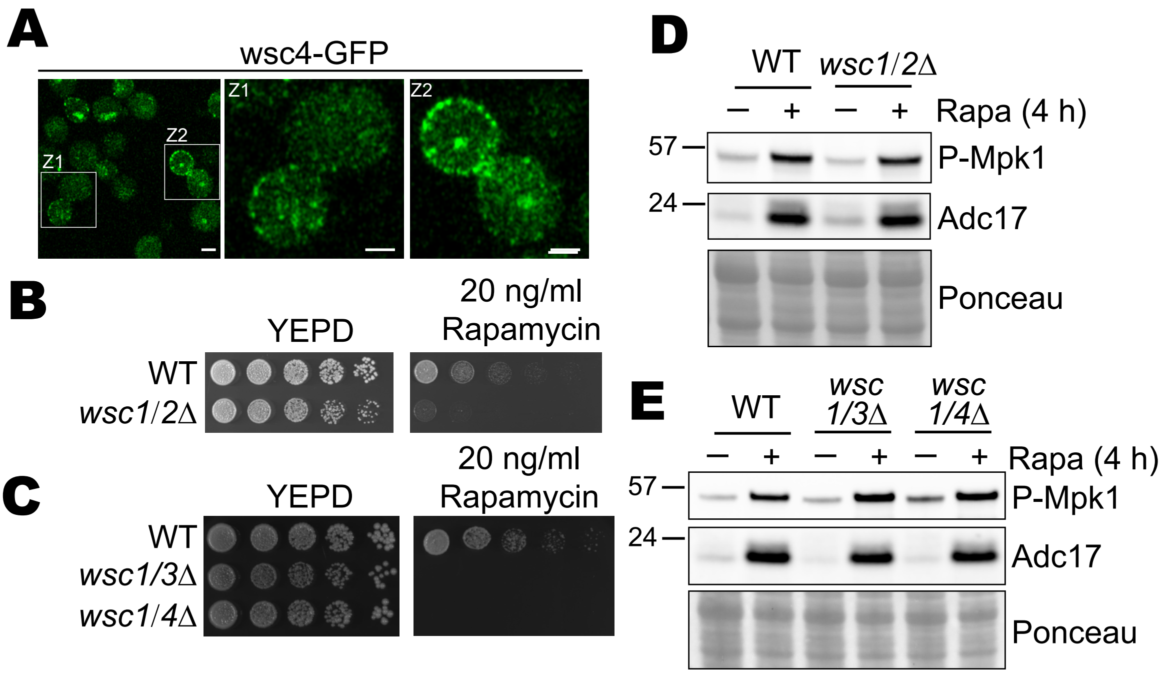

Fig. S1. Related to Figure 1. Roles of CWI sensors in the response to rapamycin.
A) Localisation of endogenously tagged Wsc4-GFP. Representative of 3 experiments.
B) Drop assay of WT and *wsc1/2*Δ cells on YEPD and YEPD + rapamycin after 3 days of growth. Representative of 3 experiments.
C) Drop assay of WT, *wsc1/3*Δ and *wsc1/4*Δ cells on YEPD and YEPD + rapamycin after 3 days of growth. Representative of 3 experiments.
D) Mpk1 activation (P-Mpk1) and Adc17 levels following 4 h rapamycin treatment in WT and *wsc1/2*Δ cells. Representative of 3 experiments.
E) Mpk1 activation (P-Mpk1) and Adc17 levels following 4 h rapamycin treatment in WT, *wsc1/3*Δ, and *wsc1/4*Δ cells. Representative of 3 experiments.

Journal of Cell Science • Supplementary information

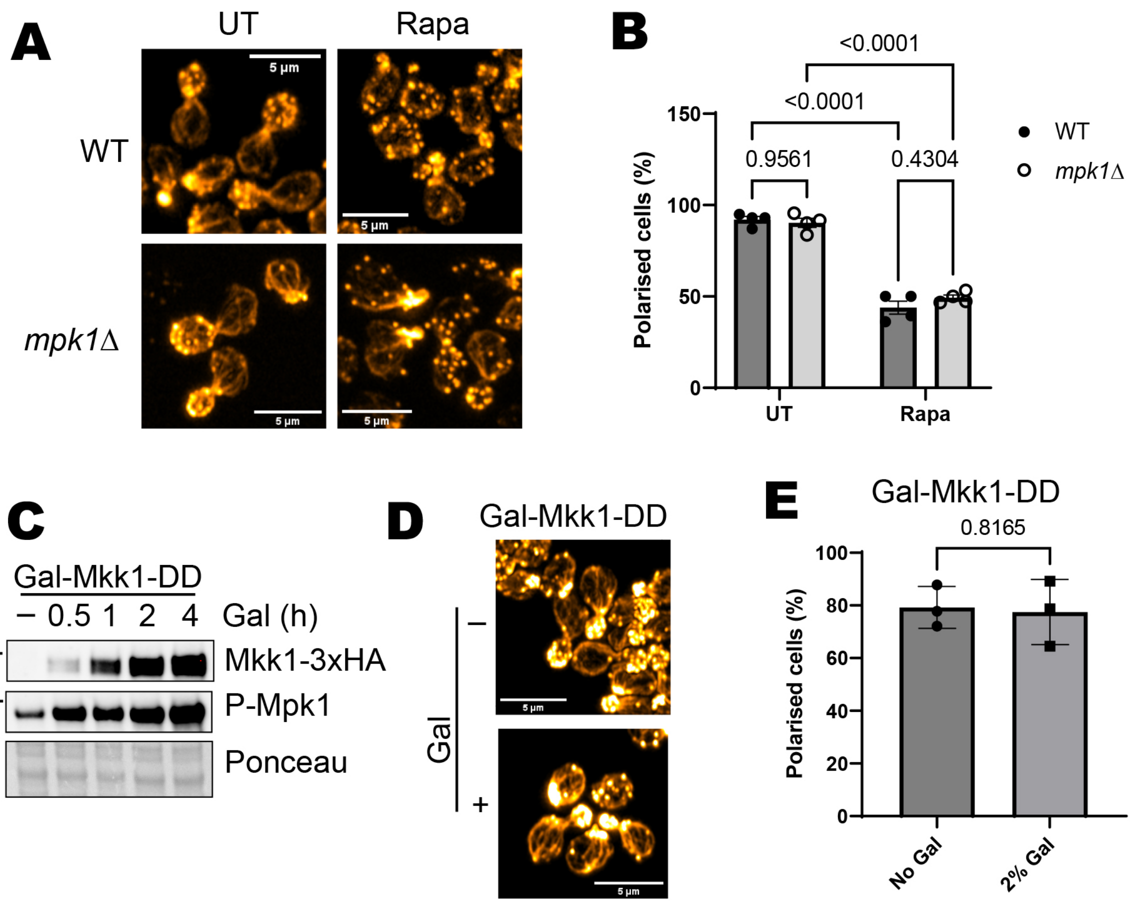

Fig. S2. Related to Figure 2. Actin depolarisation following rapamycin treatment is activated by the CWI pathway independently of Mpk1 activation.

A) Effect of 1 h rapamycin treatment on actin polarisation in WT and *mpk1*Δ cells. Representative of 4 experiments. Cellular actin is labelled with rhodamine-phalloidin. Scale bars show 5 µm.

B) Quantification of images from A (mean with SEM, n=4, two-way anova followed by a Tukey's multiple comparisons test). Budding cells are classified as unpolarised if there are 6 or more circular actin patches in the mother cell and polarised if not.

C) Induction of Mkk1-DD-3xHA from the Gal10 galactose inducible promoter, and consequent activation of Mpk1 (P-Mpk1) in WT cells. Representative of 3 experiments.

D) Effect of Mkk1-DD-3XHA induction (1 h galactose) on actin polarisation in WT cells. Representative of at least 3 experiments. Cellular actin is labelled with rhodamine-phalloidin. Scale bars show 5 µm.

E) Quantification of images from D (mean with SEM, n=3, t-test¬). Budding cells are classified as unpolarised if there are 6 or more circular actin patches in the mother cell and polarised if not.

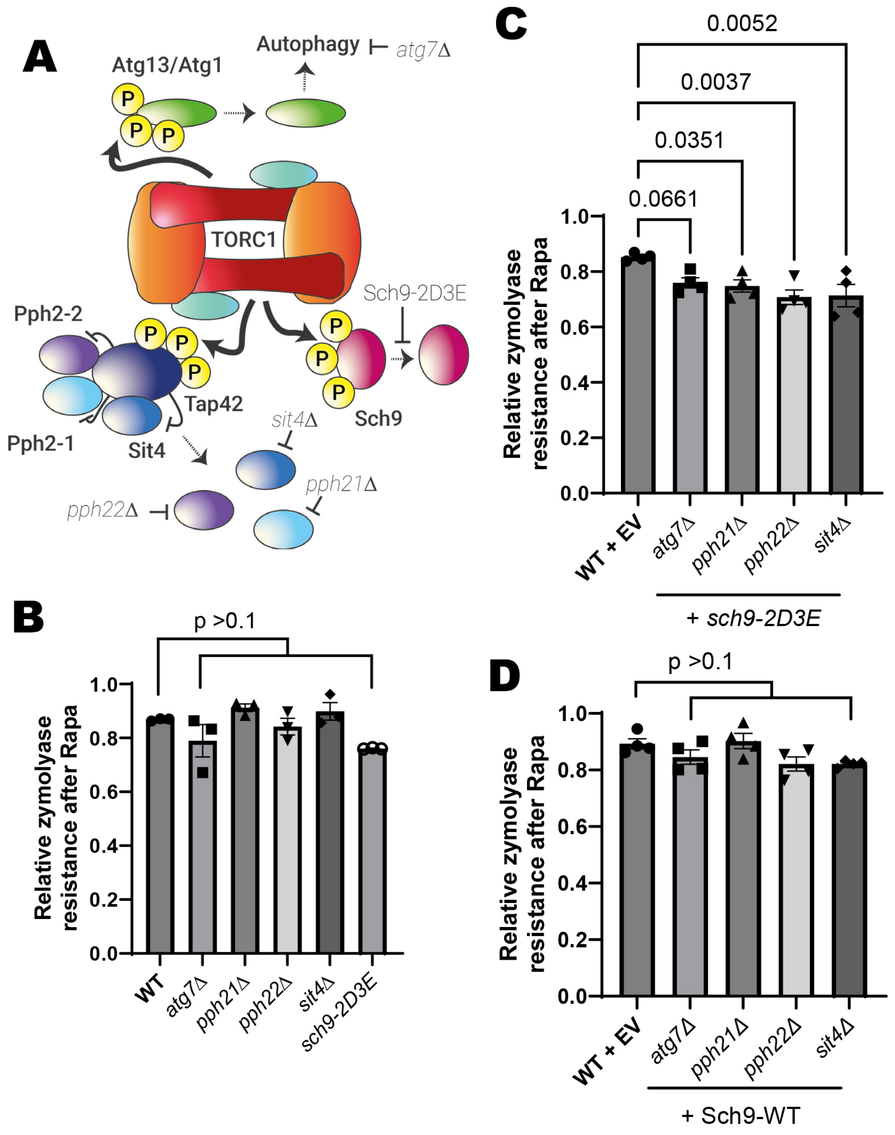

Fig. S3. Related to Figure 3.TORC1 inhibts cell wall integrity activation through multiple pathways.

A) **T**ORC1 signalling to the autophagy, Tap42/PP2A phosphatase, and Sch9 effector branches. Mutants are displayed which prevent changes to each branch upon TORC1 inhibition.
B) Resistance to 45 min zymolyase treatment after 1 h rapamycin treatment in the mutants indicated in Figure S2A (mean with SEM, n=3, one-way anova followed by a Tukey's multiple comparisons test.

C) Resistance to 45 min zymolyase treatment after 1 h rapamycin treatment in the mutants indicated in Figure S2A expressing Sch9-2D3E (mean with SEM, n=4, one-way anova followed by a Tukey's multiple comparisons test).
D) Resistance to 45 min zymolyase treatment after 1 h rapamycin treatment in the mutants indicated in Figure S2A expressing Sch9-WT (mean with SEM, n=4, one-way anova followed by a Tukey's multiple comparisons test).

Journal of Cell Science • Supplementary information

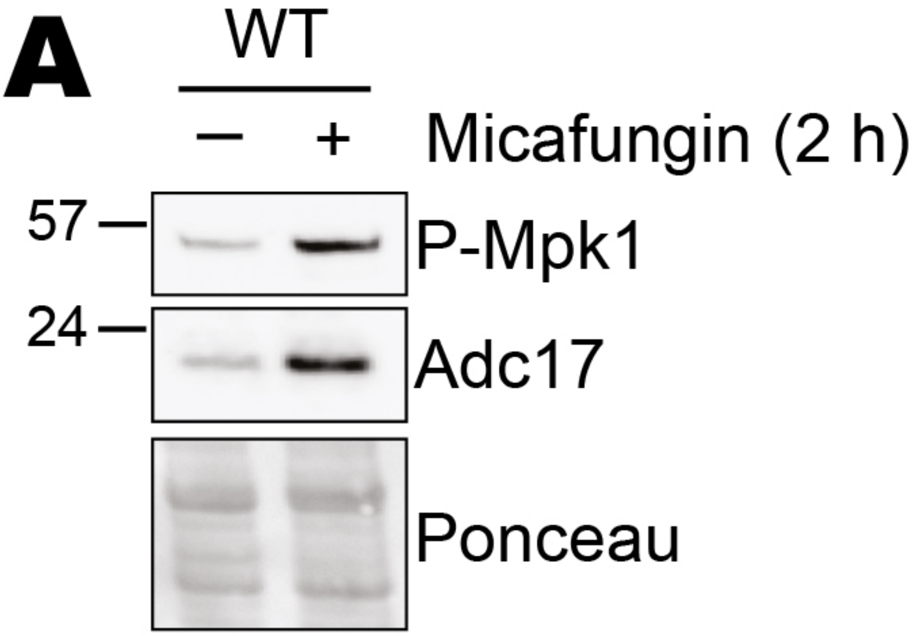

Fig. S4. Related to Figure 4. Effect of Micafungin treatment on Mpk1 activation and Adc17 protein production.

A) Mpk1 activation (P-Mpk1) and Adc17 protein levels in WT and *mpk1*Δ cells after 2 h Micafungin treatment. Representative of 3 experiments.

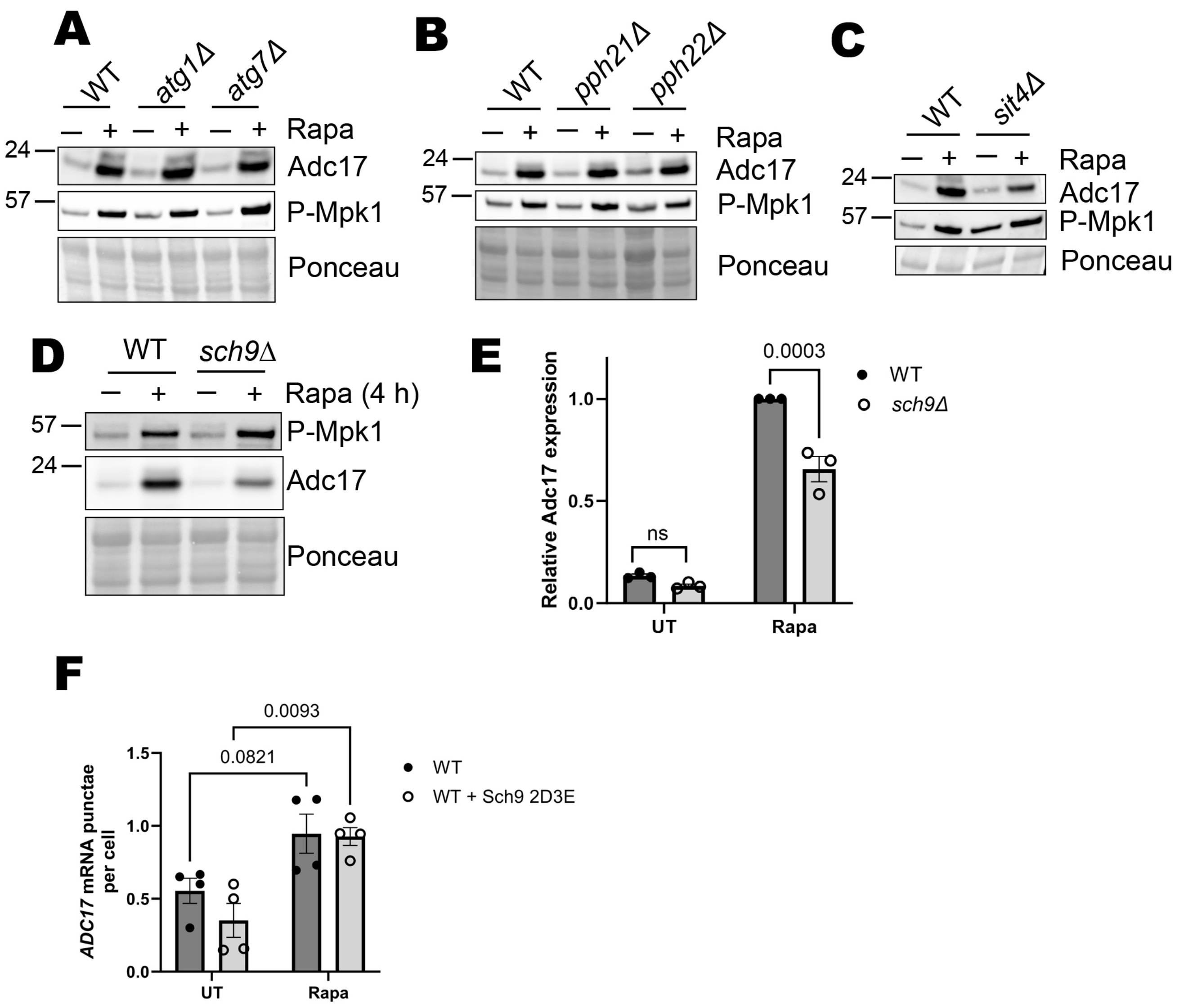

Fig. S5. Related to Fig. 6. Effect of removing proteins regulated by TORC1 on Mpk1 activation and Adc17 production following TORC1 inhibition.

A) Mpk1 activation (P-Mpk1) and Adc17 levels in WT and the autophagy deficient *atg1*Δ and *atg7*Δ mutants after 4 h rapamycin treatment. Representative of 3 experiments.
B) Mpk1 activation (P-Mpk1) and Adc17 levels in WT, *pph21*Δ, and *pph22*Δ mutants after 4 h rapamycin treatment. Representative of 3 experiments.
C) Mpk1 activation (P-Mpk1) and Adc17 levels in WT and *sit4*Δ cells after 4 h rapamycin treatment. Representative of 3 experiments.
D) Mpk1 activation (P-Mpk1) and Adc17 levels in WT and *sch9*Δ cells after 4 h rapamycin treatment. Representative of 3 experiments.
E) Quantification of Adc17 protein expression from blots shown in D (mean with SEM, n=3, two-way anova followed by a Tukey's multiple comparisons test).
F) Quantification of average number of ADC17 mRNA punctae observed per cell from Fig. 6C (mean with SEM, n=4, two-way anova followed by a Tukey's multiple comparisons test).

Journal of Cell Science • Supplementary information

Fig. 1.

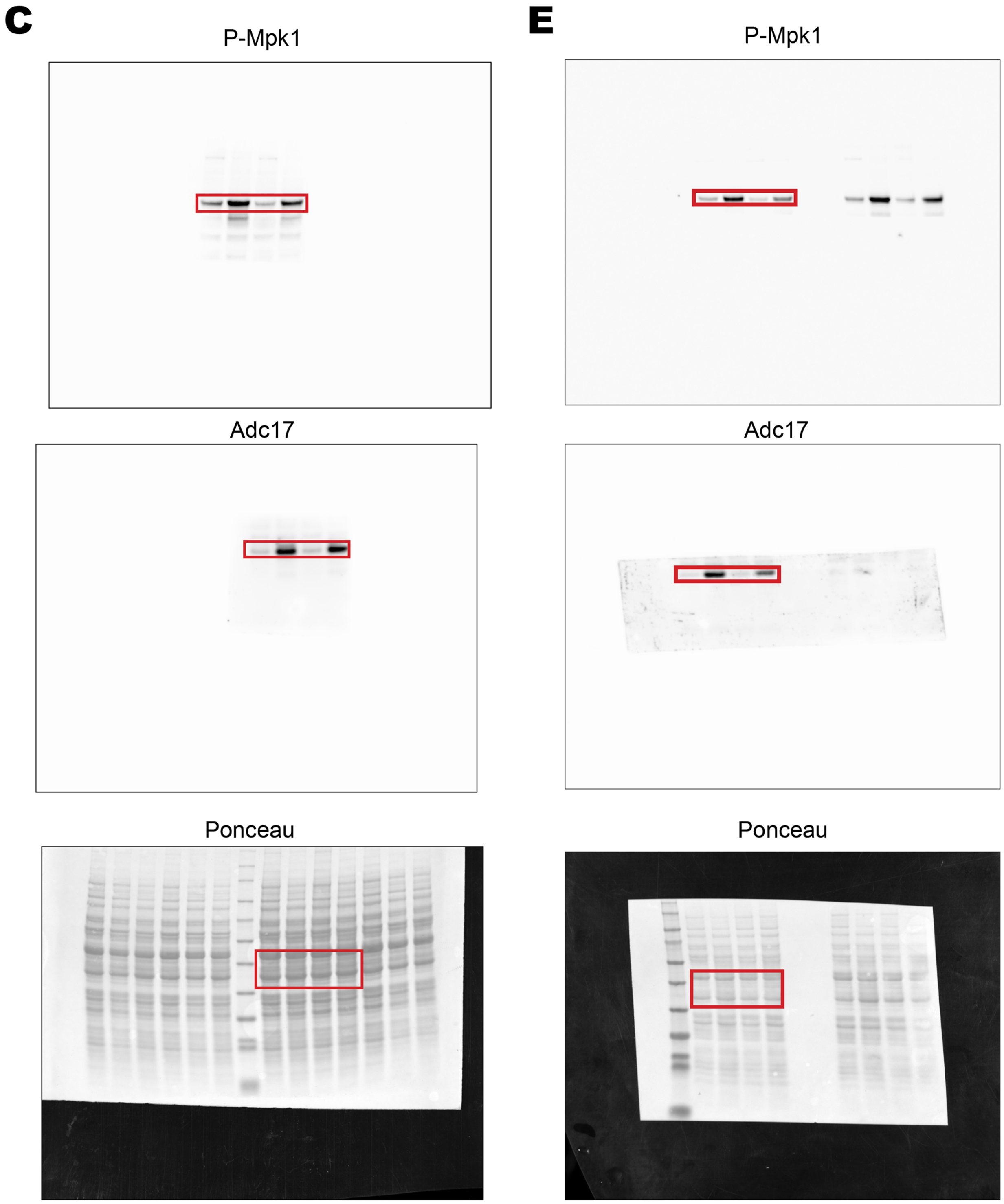

Journal of Cell Science • Supplementary information

Fig. 4:

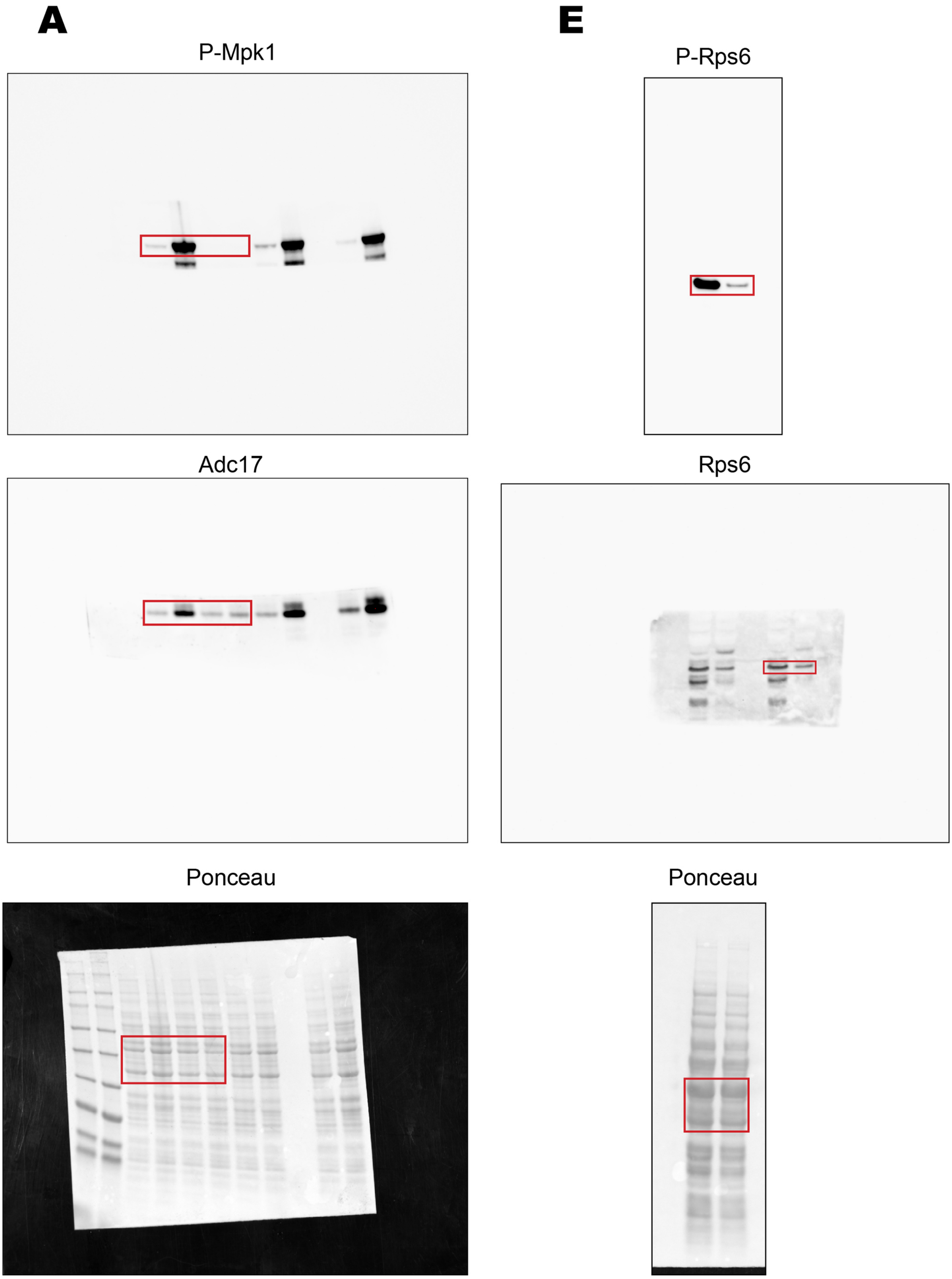

Journal of Cell Science • Supplementary information

Fig. 5:

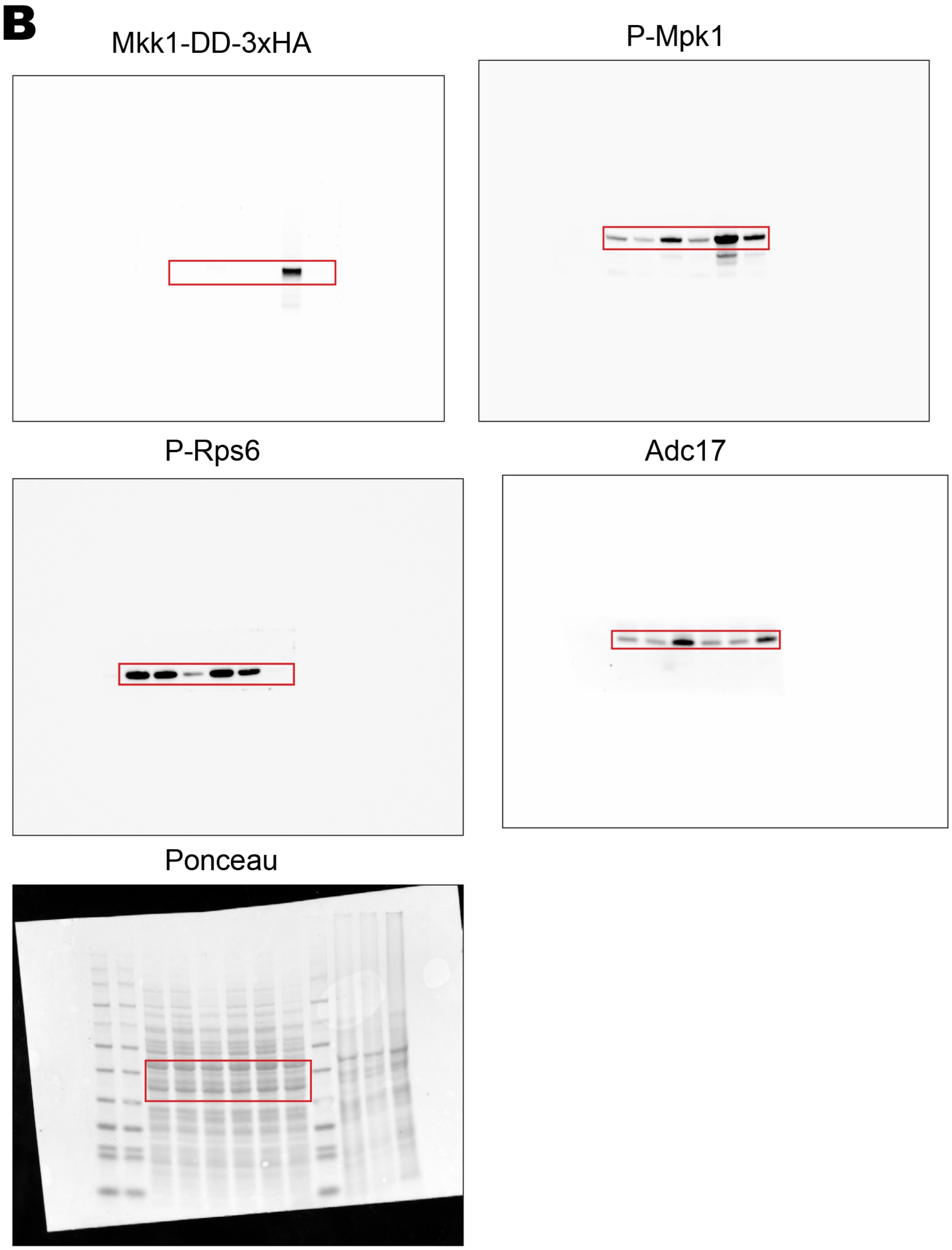

Journal of Cell Science • Supplementary information

Fig. 5:

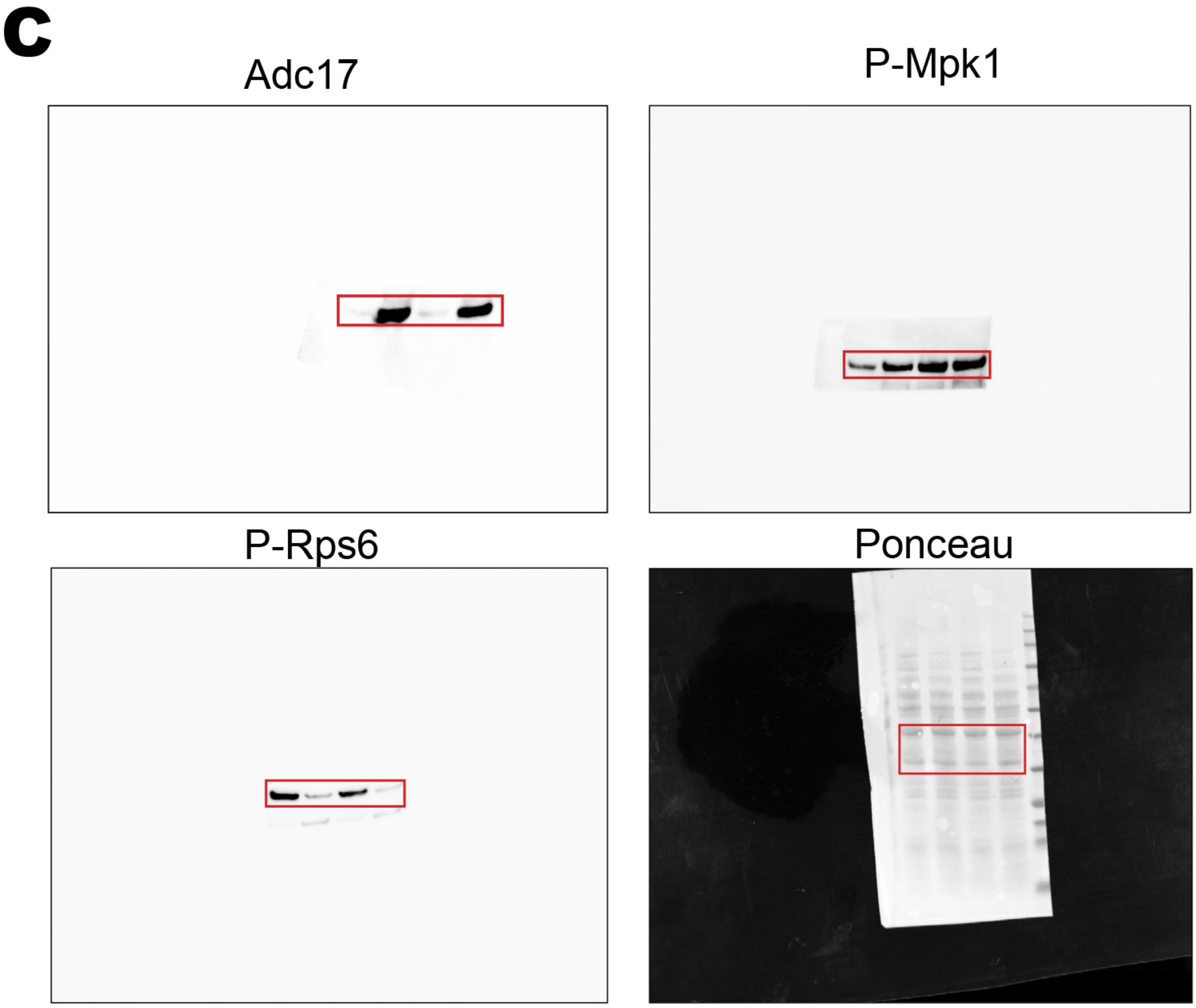

Journal of Cell Science • Supplementary information

Fig. 6:

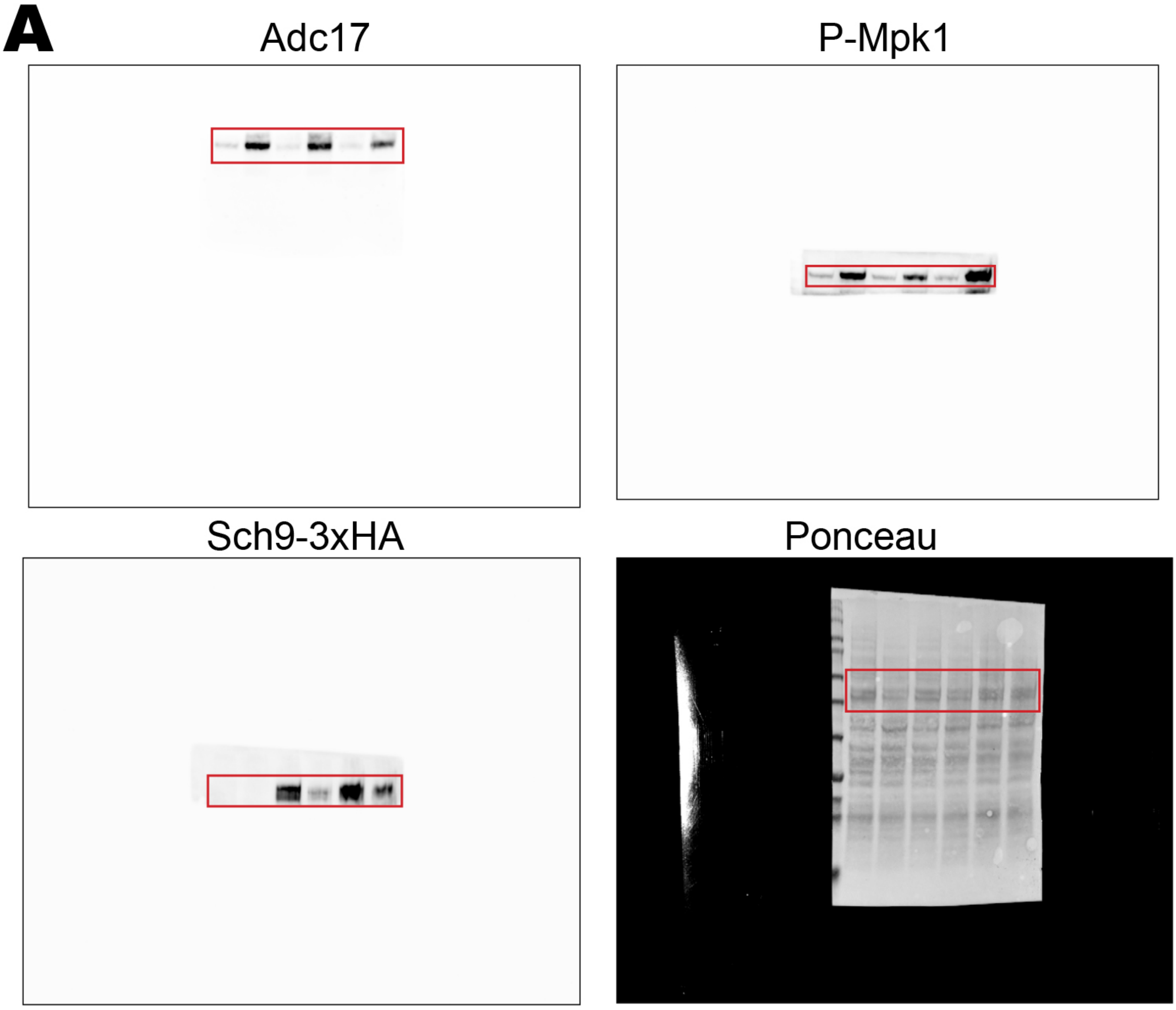

Journal of Cell Science • Supplementary information

Fig. 6:

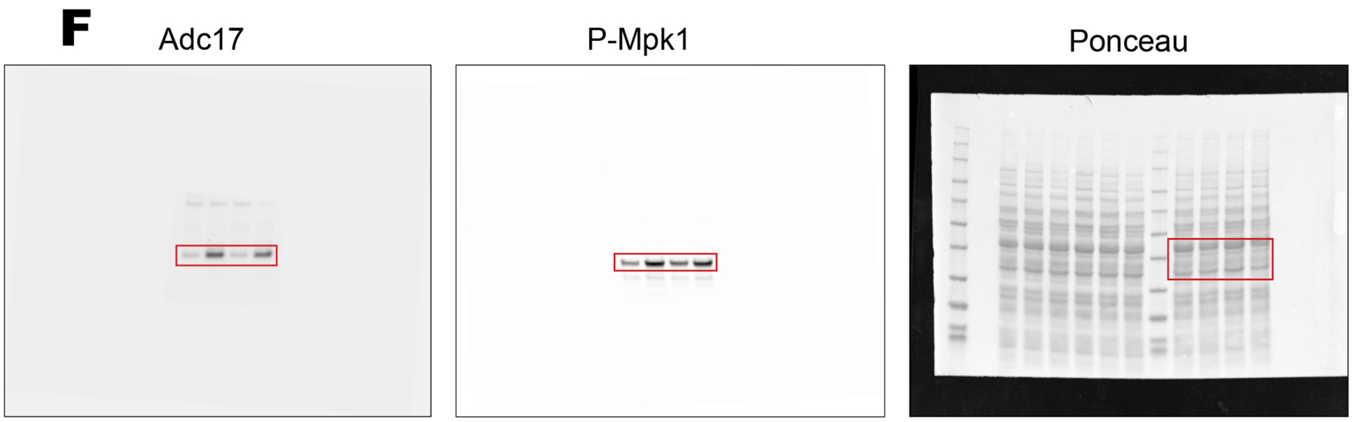

Fig. 7:

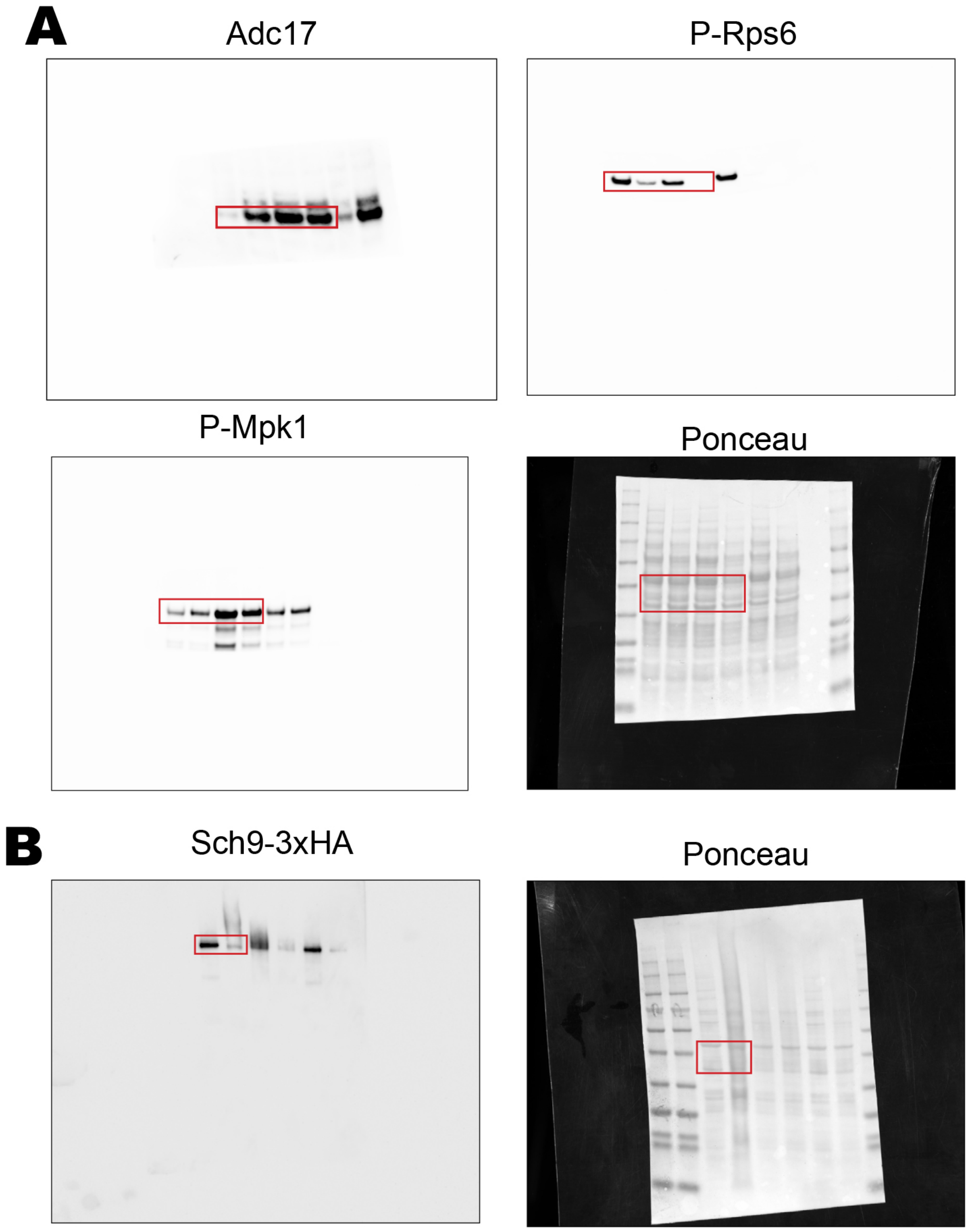

Journal of Cell Science • Supplementary information

Fig. 7:

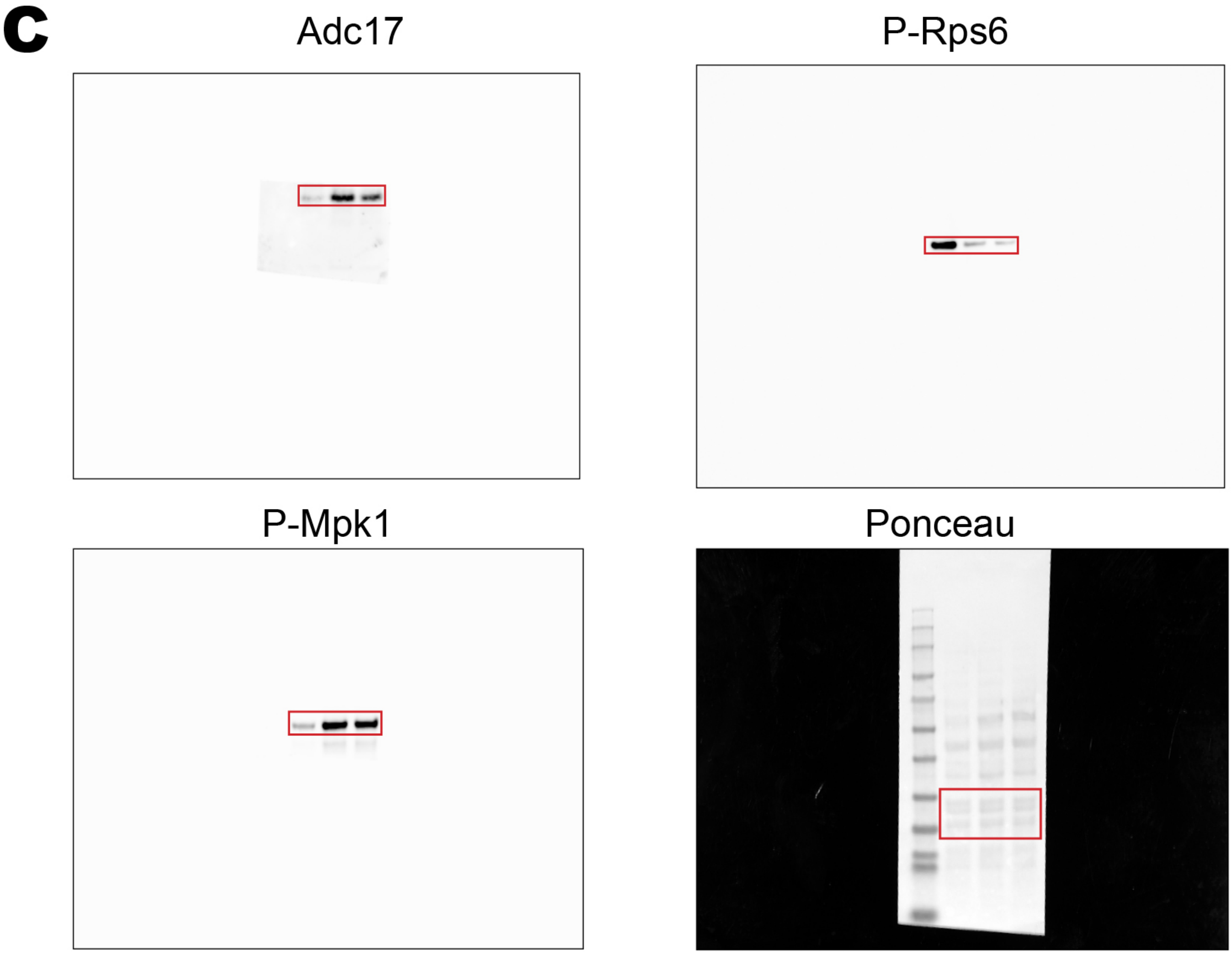

Journal of Cell Science • Supplementary information

Fig. S1:

D

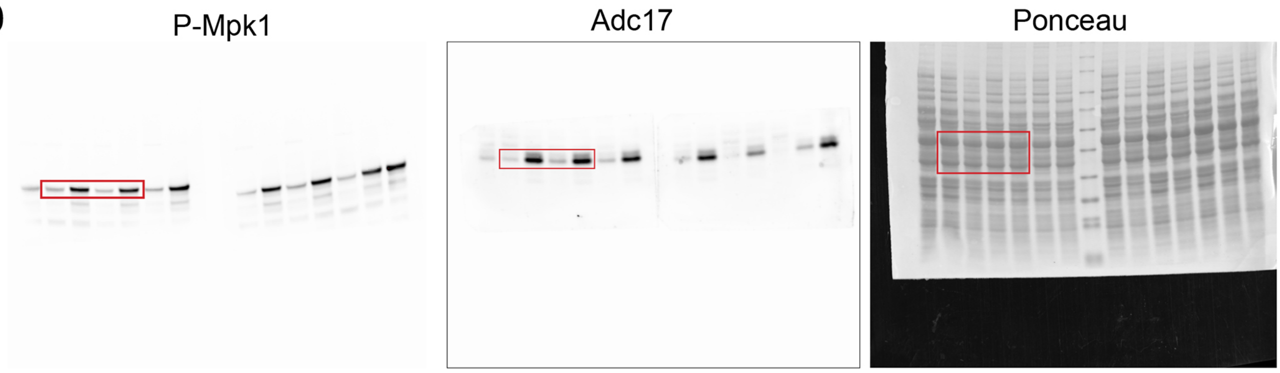

E

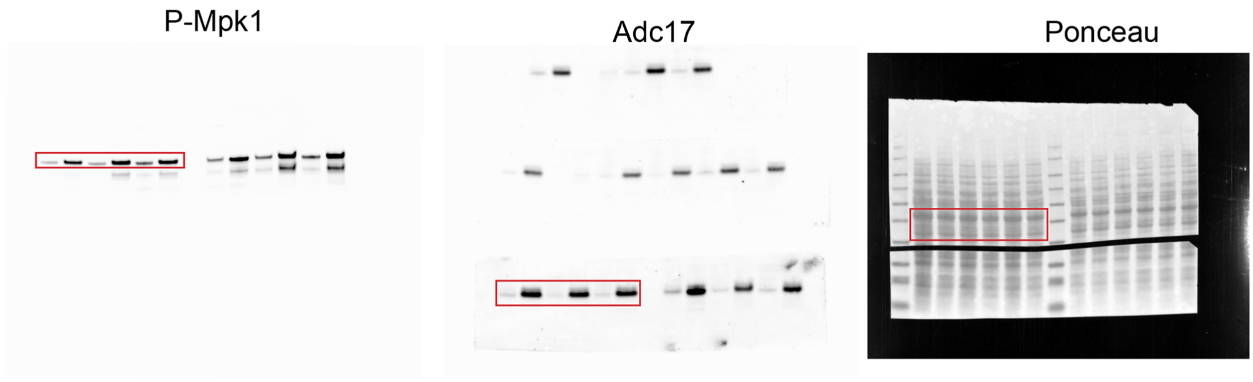

Fig. S2:

C

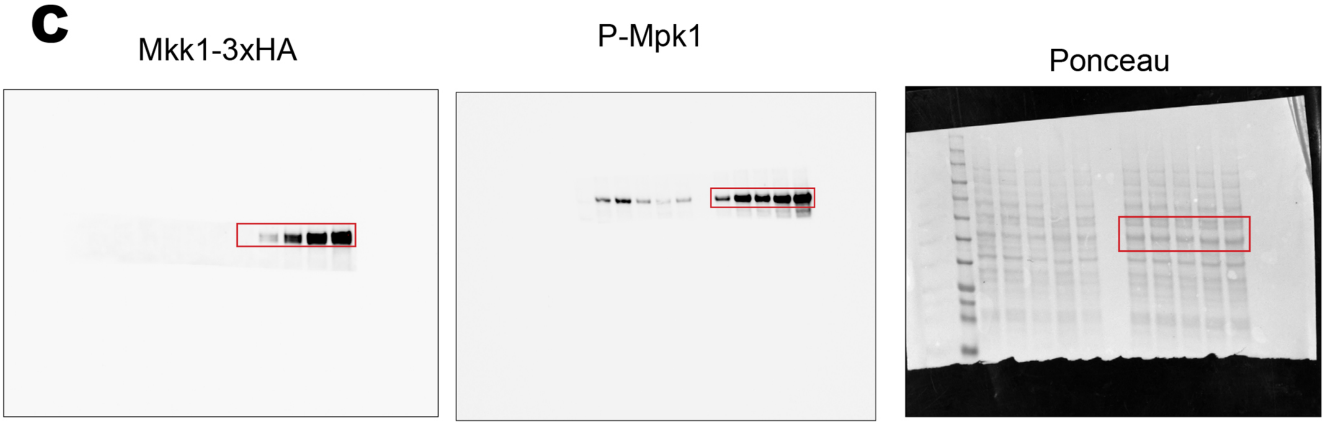

Journal of Cell Science • Supplementary information

Fig. S4:

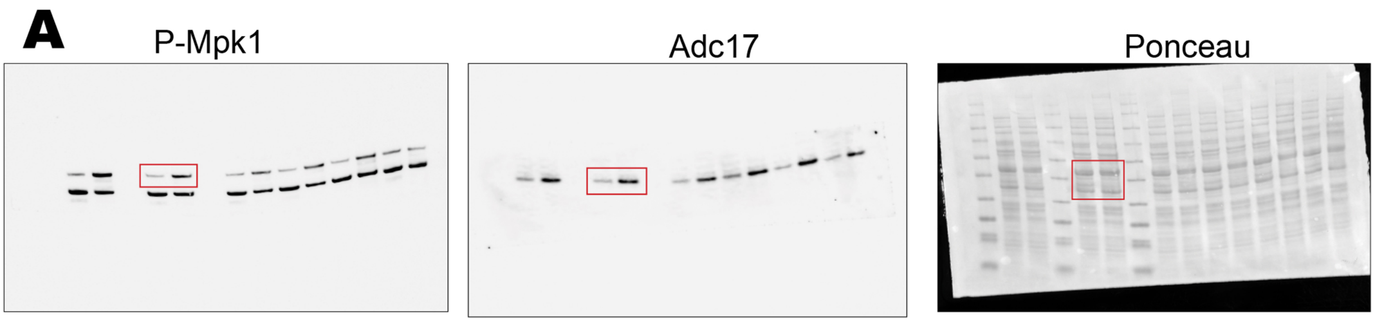

Fig. S5:

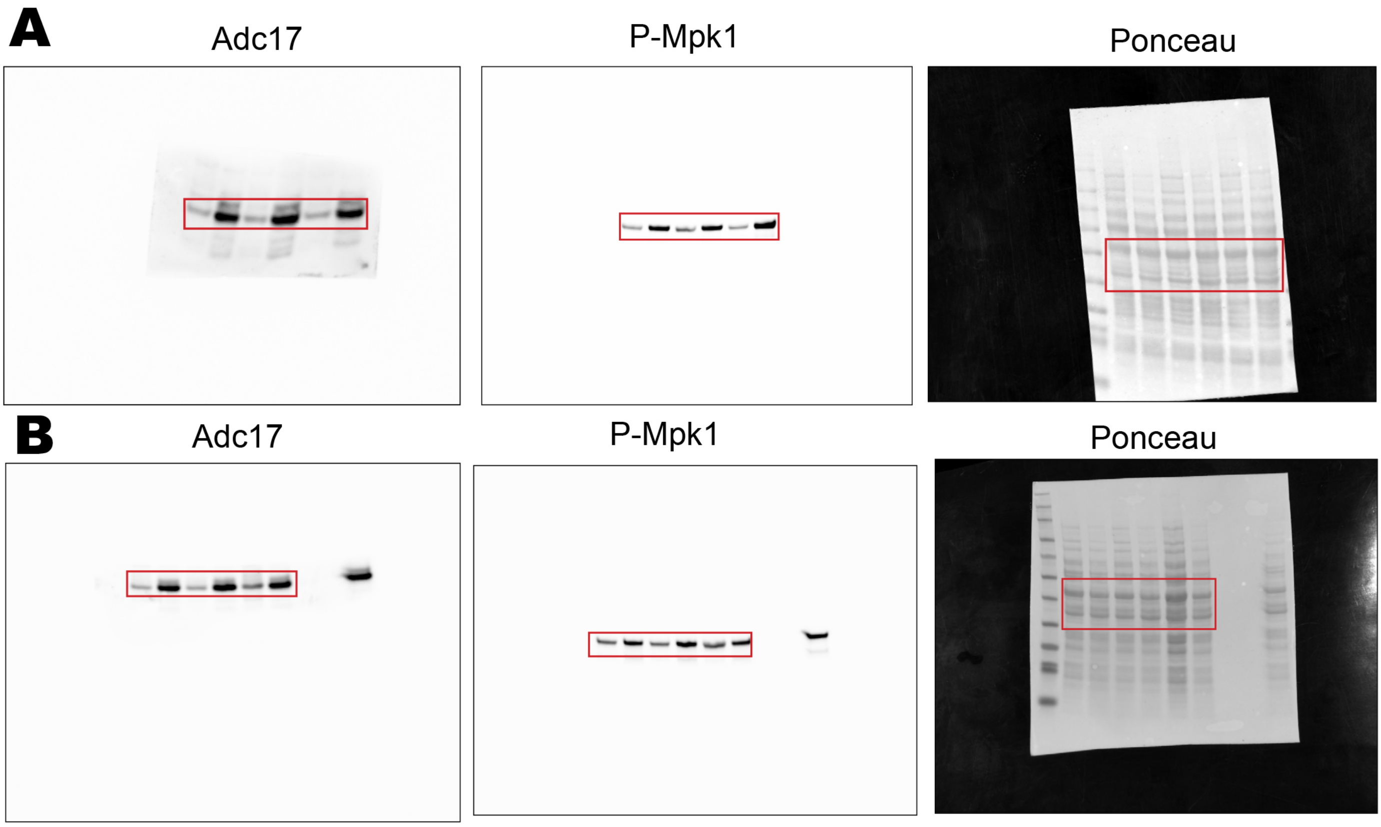

Journal of Cell Science • Supplementary information

Fig. S5:

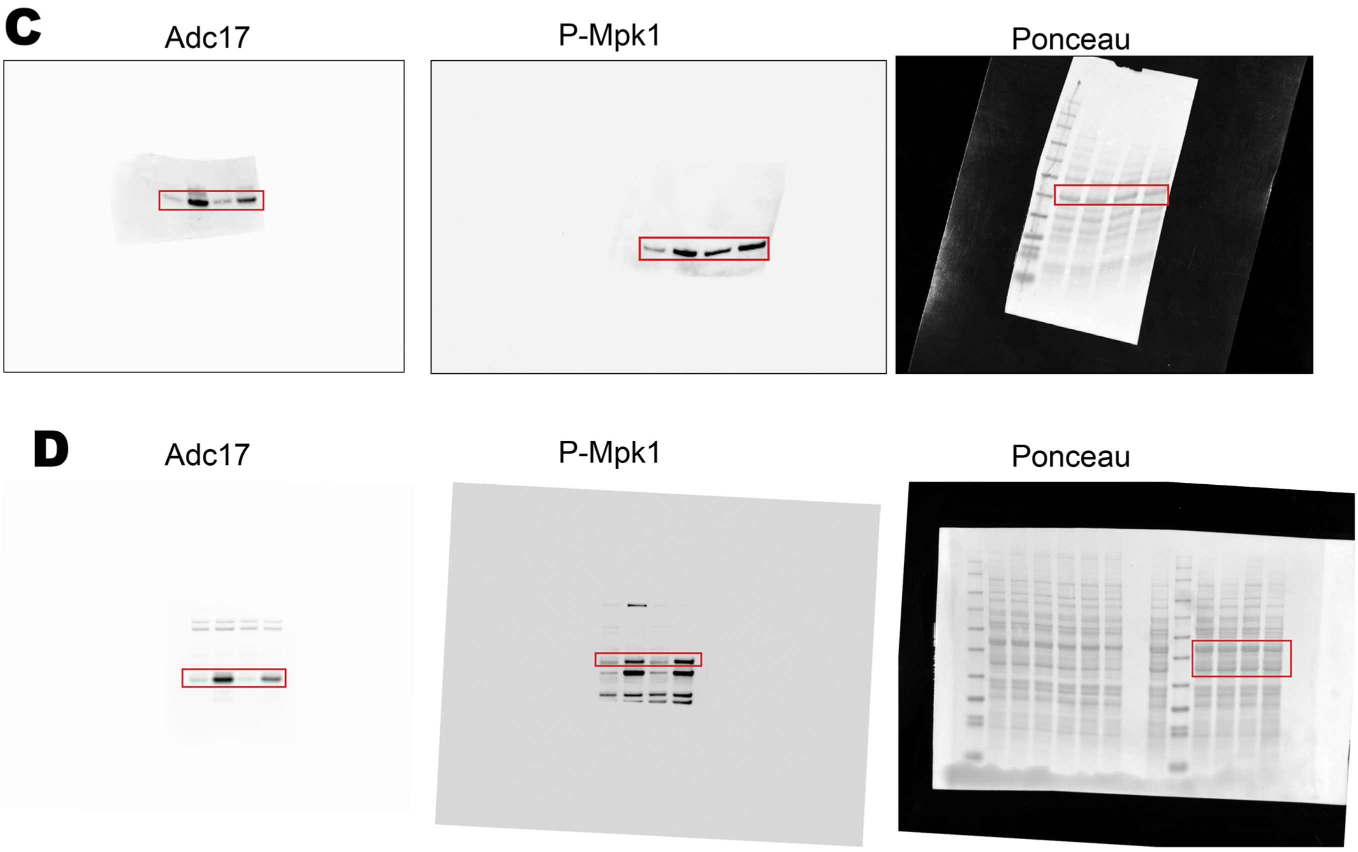

Fig. S6. Blot transparency igure. Full membranes, and the sections used in this manuscript (red boxes), are shown here.

Journal of Cell Science • Supplementary information

Table S1. List of strains used in this article and information about their origin. Strains and vectors can be obtained by request from TDW or AR.

Strain	Source
BY4741 (WT)	Horizon Discovery
BY4741 *wsc1Δ::KanMX*	Horizon Discovery
BY4741 *wsc2Δ::KanMX*	Horizon Discovery
BY4741 *wsc3Δ::KanMX*	Horizon Discovery
BY4741 *wsc4Δ::KanMX*	Horizon Discovery
BY4741 *mid2Δ::KanMX*	Horizon Discovery
BY4741 *mtl1Δ::KanMX*	Horizon Discovery
BY4741 *wsc1Δ::KanMX wsc2Δ::His3*	This study
BY4741 *wsc1Δ::Leu2 wsc3Δ::KanMX*	This study
BY4741 *wsc1Δ::Leu2 wsc4Δ::KanMX*	This study
BY4741 *wsc4-GFP::KanMX*	This study
BY4741 *wsc1Δ::Leu2 wsc3Δ::Ura3 wsc4Δ::KanMX*	This study
BY4741 *mpk1Δ::KanMX*	Horizon Discovery
BY4741 *pESC-Mkk1(S377D,T381D)-3xHA::Leu2*	This study
BY4741 *atg7Δ::KanMX*	Horizon Discovery
BY4741 *pph21Δ::KanMX*	Horizon Discovery
BY4741 *pph22Δ::KanMX*	Horizon Discovery
BY4741 *sit4Δ::KanMX*	Horizon Discovery
BY4741 *p416-*	This study
BY4741 *atg7Δ::KanMX p416-*	This study
BY4741 *pph21Δ::KanMX p416-*	This study
BY4741 *pph22Δ::KanMX p416-*	This study
BY4741 *sit4Δ::KanMX p416-*	This study
BY4741 *atg7Δ::KanMX p416-Sch9-3xHA::Ura3*	This study
BY4741 *pph21Δ::KanMX p416-Sch9-3xHA::Ura3*	This study
BY4741 *pph22Δ::KanMX p416-Sch9-3xHA::Ura3*	This study
BY4741 *sit4Δ::KanMX p416-Sch9-3xHA::Ura3*	This study
BY4741 *adc17-24xSL::KanMX abp1-aGFP::Leu2 pFA6-Cyc1pr-PP7-2xeGFP::His3 pESC::Ura3*	This study
BY4741 *adc17-24xSL::KanMX abp1-aGFP::Leu2 pFA6-Cyc1pr-PP7-2xeGFP::His3*	This study
BY4741 *adc17-24xSL abp1-mKate2-aGFP::Leu2*	Williams et al., 2022
BY4741 *adc17-24xSL abp1-mKate2-aGFP::Leu2 ede1Δ::Ura3 pFA6-Cyc1pr-PP7-*	Williams et al., 2022
BY4741 *p416::Ura3*	This study
BY4741 *p416-Sch9-3xHA::Ura3*	This study
BY4741 *adc17-24xSL::KanMX pFA6-*	This study
BY4741 *sch9Δ::KanMX*	Horizon Discovery
BY4741 *wsc1Δ::Leu2 wsc3Δ::Ura3 wsc4Δ::KanMX p416-*	This study
BY4741 *chc1Δ::KanMX*	Horizon Discovery
BY4741 *chc1Δ::KanMX p416-Sch9-3xHA::Ura3*	This study
BY4741 *chc1Δ::KanMX p416::Ura3*	This study
BY4741 *chc1Δ::KanMX p416-*	This study
BY4741 *pFA6-Cyc1pr-PP7-2xeGFP::His3 p416-*	This study
BY4741 *chc1Δ::KanMX pFA6-Cyc1pr-PP7-2xeGFP::His3 p416-Adc17-24xSL::Ura3*	This study

Journal of Cell Science • Supplementary information
