# Supplementary material for: Distinct TORC1 signalling branches regulate Adc17 proteasome assembly chaperone expression
Source: J Cell Sci. 2024 Jul 22;137(14):jcs261892. doi: 10.1242/jcs.261892 (PMC11298713; doi:10.1242/jcs.261892)
Supplement: Supplementary file 1 [file joces-137-261892_review_history.pdf]

# Distinct TORC1 signalling branches regulate Adc17 proteasome assembly chaperone expression

Thomas D. Williams, Ifeoluwapo Joshua, Flavie Soubigou, Sylwia M. Dublanska, Rebecka Bergquist and Adrien Rousseau
DOI: 10.1242/jcs.261892

**Editor**: Michael Way

**Review timeline**

| | |
|---|---|
| Original submission: | 12 December 2023 |
| Editorial decision: | 29 January 2024 |
| First revision received: | 3 June 2024 |
| Accepted: | 24 June 2024 |

---

**Original submission**

First decision letter

MS ID#: JOCES/2023/261892

MS TITLE: Distinct TORC1 signalling branches regulate Adc17 proteasome assembly chaperone expression

AUTHORS: Thomas David Williams, Rebecka bergquist, Sylwia Dublanska, and Adrien Rousseau
ARTICLE TYPE: Research Article

We have now reached a decision on the above manuscript.

To see the reviewers' reports and a copy of this decision letter, please go to: https://submit-jcs.biologists.organd click on the 'Manuscripts with Decisions' queue in the Author Area. (Corresponding author only has access to reviews.)

As you will see, all three reviewers raise a number of substantial criticisms that prevent me from accepting the paper. At this stage, the overall feeling is that the paper lacks some important controls and does not represent a sufficient advance for publication in JCS. They suggest, however, that a revised version might prove acceptable, if you can address their concerns with additional experiments and clarifications. If you think that you can deal satisfactorily with the criticisms on revision, I would be pleased to see a revised manuscript. We would then return it to the reviewers.

Please ensure that you clearly highlight all changes made in the revised manuscript. Please avoid using 'Tracked changes' in Word files as these are lost in PDF conversion.

I should be grateful if you would also provide a point-by-point response detailing how you have dealt with the points raised by the reviewers in the 'Response to Reviewers' box. Please attend to all of the reviewers' comments. If you do not agree with any of their criticisms or suggestions please explain clearly why this is so.

Reviewer 1

*Advance summary and potential significance to field*

© 2024. Published by The Company of Biologists under the terms of the Creative Commons Attribution License (https://creativecommons.org/licenses/by/4.0/).

This manuscript entitled “Distinct TORC1 signaling branches regulate Adc17 1 proteasome assembly chaperone expression.” By Thomas D. Williams et al. describes the signaling pathway involved in Adc17 translation controlling during TROC1 inhibition. They showed that the activation of the Cell Wall Integrity pathway and sch9 inhibition are both required for this phenomenon.

*Comments for the author*

In general, this study lacks sufficient advance in our knowledge expected for this journal.
They identified two pathways in Adc17 regulation, but the analysis are not deepen enough. They failed to analyze quantitatively in several critical experiments. Several critical controls were lacking. Therefore, I cannot support their conclusion.

Specific comments
1 Figure 1C. Why Mpk1 is phosphorylated in WT without rapamycin? This is critical flaw in this study. Furthermore, Mpk1 western blotting is missing throughout the study, and we cannot evaluate the phosphorylation status of Mpk1.

2 Figure 2AB. Zymolyase resistance in WT were significantly different between these two data.
3. There is no mention of FigS1E.
4. Figure S2B. Pph2-1- and 2-2 are 21 and 22. They are redundant and needed to test the double mutant.
5 Fig S2C. WT Sch9 should be expressed and compared to 2D3E.
6. FigS3E. Rps6 should be immunoblotted.
7. Fig3B. Why there is a Mkk1dd3HA signal in the cells without harboring it.
8. Fig 4B. Why Sch9 expression is quite low in chc1 mutant?
9. What is Line 109?

Reviewer 2

*Advance summary and potential significance to field*

The manuscript by Williams et al describes how TORC1 controls the expression of Adc17, the proteosome assembly chaperone. The manuscript introduces two novel signal branches to Adc17, namely the cell wall integrity pathway sensor proteins and the inhibition of Sch9. This work builds upon prior research (PMID: 35739319 and PMID: 27462806).

*Comments for the author*

The manuscript by Williams et al describes how TORC1 controls the expression of Adc17, the proteosome assembly chaperone. The manuscript introduces two novel signal branches to Adc17, namely the cell wall integrity pathway sensor proteins and the inhibition of Sch9. This work builds upon prior research efforts (PMID: 35739319 and PMID: 27462806). The presentation of data in the manuscript is generally well-executed. Below are my specific comments:

1. The expression "proteasome assembly chaperone" appears awkward; it may be worth considering a rewrite, such as "expression of the protein assembly chaperone." Additionally, consider placing Adc17 in the abstract.
2. The manuscript studies the expression of Adc17 protein levels. The main assay is western blot, and determine the difference in Adc17 levels (e.g. Figure 1E). It would be good to include some level of quantification to back up the statements related to changes in Adc17 levels, ideally with loading control other then ponceau staining.
3. “Three of the mutants, wsc1Δ, wsc3Δ, and wsc4Δ showed a mild to moderate growth defect. The most severely affected strain, wsc1Δ, has previously been implicated in Mpk1 activation following rapamycin treatment (Krause and Gray 2002). We did not observe an Mpk1 activation defect using our wsc1Δ strain and conditions however (Figure 1C).” it seems to me mtl1 also has mild phenotype. wsc1 has strong phenotype since I cannot see any growth on the rapamycin treatment.
4. “Reasoning that there may be functional redundancy between the Wsc1, Wsc3, and Wsc4 sensor proteins, we made a wsc1/3/4Δ strain. We found that wsc1/3/4Δ cells are incapable of growth in the presence of rapamycin (Figure 1D) and have greatly diminished Mpk1 activation following

© 2024. Published by The Company of Biologists under the terms of the Creative Commons Attribution License (https://creativecommons.org/licenses/by/4.0/).

rapamycin treatment (Figure 1E).” While there is perhaps some redundancy to Mpk1 signalling it is not growth assay. Maybe make it clear in 1B that is some growth left?
5. Combining these two features was insufficient to achieve Adc17 expression in the absence of rapamycin (Figure 3B). please label the lanes and refer to them when describing the data. Also describe the data in figure 3B a bit more elaborate. E.g. there is no mention on P-Rps6 control.
6. Excitingly, there was a notable decrease in Adc17 expression in Sch9-2D3E expressing cells, suggesting a role for Sch9 inhibition (Figure 3C). Please define the Sch9 mutant plus reference. I assume this is a hyperactive mutant.
What happens in Sch9 delete ?
7. There are notable questions that merit discussion and potential experimental investigation. For instance, what are the effects in the sch9D mutant?
Additionally, how do wcs/1/3/4 + Sch9 hyperactive mutants respond?
8. If I understand correctly, TORC also regulate Mpk1. If so, it seems important to depict this relationship in Figure 4E.

Reviewer 3

*Advance summary and potential significance to field*

The work of Williams et al. reports on studies aimed at defining the relations between TORC1 and CWI signalling and its effect on proteasome function. The authors report on a considerable amount of work with experiments from different viewpoints and used a variety of mutants and expression constructs of different genes. However, in contrast to what is claimed in the abstract and in results and discussion, a clear picture of what the interactions are and what they mean cannot be derived. This is partially due to some basic errors and lack of proper controls, as listed in the following:

*Comments for the author*

1. A major drawback of this study is the perception, that Wsc4 would be a cell wall sensor residing in the plasma membrane (depicted in Fig. 2G). In fact, this protein localizes at the ER and has different functions from the sensors (see for example SGD database or any of the reviews cited). Thus, there really are only 5 (and not 6) CWI sensors, as claimed in line 83.
2. In this context, and beyond, reasoning from line 90ff must be considered. First, it is not clear why the authors choose wsc1 wsc3 wsc4 triple mutants to study redundancy. From previous works it would appear that Wsc1, Wsc2, and Wsc3, in that very order, exert the important functions. It would thus have been logic to use at least the triple deletion of those.
As Wsc4 has probably no function in what the authors are studying (see above), a minimum to test would have been the double deletion wsc1 wsc3. Again, I would strongly suggest to also test wsc1 wsc2.
With this in mind, the statement “These data suggest that TORC1 inhibition activates the CWI through the Wsc1, Wsc3, and Wsc4 sensor proteins.” is clearly an unwarrented overinterpretation.
3. In line 103ff the authors test the hyperactivation of Mpk1 by a mutant allele of MKK1 expressed under an inducible GAL promoter. This means switching carbon sources, which is a problem by itself in this context. What is missing in this experiment is a control of the wild-type MKK1 allele expressed under the same promoter, which would be expected to have no effect.
4. Line 109 states “CWI-Bni1-actin polarity section deleted as suggested, ready for the reviewers if required.” – What is this? – Suggested by whom?
5. Line 132ff reports on Zymolyase sensitivity. The conclusion is an effect on the cell wall. To really conclude that (line 150ff), both cell wall carbohydrates and cell wall protein concentrations need to be compared between the strains.
6. Line 157ff: To induce CWI signalling, calcofluor white is employed as a “well described activator”. So this is true, it is also affecting cell wall signalling in a rather indirect way in interacting with chitin. The authors may want to consider repeating these experiments with a direct glucanase inhibitor of the echinocandin class.
7. Line 168: Tethering of the mRNA to actin should be explained in more detail. How is this done? – Is there proof that tethering does not interfere with translation efficiency?
8. Interaction of Mpk1 activation with TORC1/Sch9 signalling: I think the small GTPase Rho5 was shown to work as a negative regulator of CWI signalling and also shows synthetic lethality with

© 2024. Published by The Company of Biologists under the terms of the Creative Commons Attribution License (https://creativecommons.org/licenses/by/4.0/). 

sch9 deletions? – The authors may want to consider this as the unknown “additional factor”? – At least it merits discussion.
9. The authors speak frequently of ADC17 expression. Expression is a term that should be reserved to transcription of genes, but is often confused with the amount/production of proteins by biochemists in the literature. Especially in this manuscript, this practice is missleading, as it becomes clear only by careful reading of the text, that what is meant is the translation of pre-formed mRNA, rather than transcription. Phrasing should thus make clear if they talk about gene expression, translation efficiency, or protein production/concnetration.
10. Methods: line 322 “Cells were grown overnight ...” – Is there any way the authors made sure that cells are still growing logarithmically and were in the same growth phase for comparisons? – At least OD600 should be determined.
line 342ff: surely cells were not washed with 600/400 ml of buffer etc. Please correct for microliters line 348: likewise, 30 mg of protein would be hard to lead on a gel. I suspect that 30 microgramms of total protein are meant?
line 364ff: is this an accepted method to determine Zymolyase sensitivity? – If so, please give a reference.
In summary, while the authors performed an admirable amount of work, for this reviewer the actual progress in the field does not become evident. Where there are clear conclusions drawn into this direction, they do not seem to be warrented by the experimental data.

---

**First revision**

Author response to reviewers' comments

Dear Journal of Cell Science editorial team,

We thank the reviewers and the editors for the constructive feedback. We believe we have now satisfied the reviewers points, and have a much stronger research article. For help with these revisions, we have added extra authors. We hope both the editors and reviewers will agree with us that this had improved the legibility of the manuscript. The specific points addressing the reviewer comments are below:

Reviewer 1 Advance Summary and Potential Significance to Field:
This manuscript entitled “Distinct TORC1 signaling branches regulate Adc17 1 proteasome assembly chaperone expression.” By Thomas D. Williams et al. describes the signaling pathway involved in Adc17 translation controlling during TROC1 inhibition. They showed that the activation of the Cell Wall Integrity pathway and sch9 inhibition are both required for this phenomenon.

Reviewer Comments for the Author:
In general, this study lacks sufficient advance in our knowledge expected for this journal. They identified two pathways in Adc17 regulation, but the analysis are not deepen enough. They failed to analyze quantitatively in several critical experiments. Several critical controls were lacking. Therefore, I cannot support their conclusion.

Specific comments
1 Figure 1C. Why Mpk1 is phosphorylated in WT without rapamycin? This is critical flaw in this study. Furthermore, Mpk1 western blotting is missing throughout the study, and we cannot evaluate the phosphorylation status of Mpk1.
We have not performed Western blots of Mpk1 in this study, as levels of total and phosphorylated Mpk1 are directly related, due to phosphorylated Mpk1 activating the expression of Mpk1. Moreover, the activation of the CWI pathway is directly dictated by the levels of activated Mpk1 (p-Mpk1). We have now noted this critical point at line 45:

“Activated Mpk1 then acts to increase Mpk1 protein abundance, thus amplifying the CWI signalling

© 2024. Published by The Company of Biologists under the terms of the Creative Commons Attribution License (https://creativecommons.org/licenses/by/4.0/). 

response (García et al., 2016)."

We had originally used an over-exposed blot for Figure 1C and have now replaced it with one which was not.

2 Figure 2AB. Zymolyase resistance in WT were significantly different between these two data.

We agree that there are some minor differences, likely due to using different aliquots of the zymolyase. Importantly, the pattern between these experiments is consistent both with each other and with previous reports (e.g. Torres et al., 2002).

3. There is no mention of FigS1E.

Figure S1E is now Figure S2E and is mentioned in the text:

"Activating Mpk1 by inducible expression of a constitutively active version of the upstream kinase Mkk1, Mkk1$^{S377D;T381D}$ (Mkk1-DD) (Harrison et al., 2004), likewise had no impact on actin polarity (Figure S2C-E)."

4. Figure S2B. Pph2-1- and 2-2 are 21 and 22. They are redundant and needed to test the double mutant.

We have changed Pph2-1 and Pph2-2 to Pph21 and Pph22 respectively throughout the text and figures.

The reviewer is correct to highlight the overlapping functions of Pph21 and PPh22, and it would be good to test this. However, the double knockout is either synthetically lethal or extremely sick. We are not confident in the isolation of such a mutant without picking up suppressor mutations which may alter the phenotypes examined. Even if such a mutant were obtained with a high degree of confidence, the much slower growth observed is very likely to alter either TORC1 activity, Mpk1 activity and/or the cell wall structure, making the interpretation of any phenotype challenging. An alternative of using temperature sensitive mutants is not possible, as this adds an additional stress which impinges on TORC1 activity. We have not performed this experiment, but have noted some of these problems and the point raised by the reviewer at line 164:

"While it is established that Pph21 and Pph22 have similar, but not entirely overlapping, functions, the double mutant has either a substantial growth defect or is non-viable (Jonasson et al., 2016; Mitchell and Sprague, 2001; Shen et al., 2014). As reduced growth rates cause changes to the cell wall structure (McMurrough and Rose, 1967) and facilitates selection for suppressor mutants, we did not pursue whether deletion of both proteins may cause a stronger defect."

5 Fig S2C. WT Sch9 should be expressed and compared to 2D3E.

Figure S2C is now Figure S3C. The suggested comparison has now been made in Figure S3D.

6. FigS3E. Rps6 should be immunoblotted.

We have performed this experiment and included it in line 183:

"However, TORC1-dependent phosphorylated Rps6 (P-Rps6) was reduced following CFW treatment (Figure 4E), possibly through the action of Rho1 (Yan et al., 2012). This could not be explained by a reduction in Rps6 protein."

© 2024. Published by The Company of Biologists under the terms of the Creative Commons Attribution License (https://creativecommons.org/licenses/by/4.0/).

7. Fig3B. Why there is a Mkk1dd3HA signal in the cells without harboring it.

Having reviewed this figure, we mistakenly used a replicate where the lanes were loaded differently. We thank the reviewer for catching this and have replaced it with an updated version with the correct sample order. This is now Figure 5B.

8. Fig 4B. Why Sch9 expression is quite low in chc1 mutant?

We believe this is because reduced TORC1 activity results in lower Sch9 levels. We observed this, but had not included it in the original submission, for cells expressing Sch9 treated with rapamycin. We have now included this data in Figure 6A and discussed it from Line 243:

"We observed very low levels of Sch9 in *chc1Δ* cells, confirming that its overall function is decreased in *chc1Δ* cells compared to WT cells (Figure 7B). This could be due to the reduction in TORC1 activity, as rapamycin treatment also caused reduced levels of Sch9 (Figure 6A)."

9. What is Line 109?

Line 109 has been deleted. The data it referred has been deleted from the paper to remove complexity and improve the flow of the story.

*****

Reviewer 2 Advance Summary and Potential Significance to Field:
The manuscript by Williams et al describes how TORC1 controls the expression of Adc17, the proteosome assembly chaperone. The manuscript introduces two novel signal branches to Adc17, namely the cell wall integrity pathway sensor proteins and the inhibition of Sch9. This work builds upon prior research (PMID: 35739319 and PMID: 27462806).

Reviewer 2 Comments for the Author:
The manuscript by Williams et al describes how TORC1 controls the expression of Adc17, the proteosome assembly chaperone. The manuscript introduces two novel signal branches to Adc17, namely the cell wall integrity pathway sensor proteins and the inhibition of Sch9. This work builds upon prior research efforts (PMID: 35739319 and PMID: 27462806). The presentation of data in the manuscript is generally well-executed. Below are my specific comments:

1. The expression "proteasome assembly chaperone" appears awkward; it may be worth considering a rewrite, such as "expression of the protein assembly chaperone." Additionally, consider placing Adc17 in the abstract.

We have edited the abstract as suggested:

"Increased proteasome assembly is dependent on increased production of proteasome assembly chaperones. In *S. cerevisiae*, inhibition of the growth-promoting kinase complex TORC1 causes increased proteasome assembly chaperone translation, including that of Adc17."

2. The manuscript studies the expression of Adc17 protein levels. The main assay is western blot, and determine the difference in Adc17 levels (e.g. Figure 1E). It would be good to include some level of quantification to back up the statements related to changes in Adc17 levels, ideally with loading control other then ponceau staining.

We have used ponceau as a loading control as this uses multiple proteins, which avoids the potential pitfalls of using individual proteins whose levels can be altered by cellular stress. As suggested by the reviewer, we have now added quantifications based on this for key figures where we claim a difference in Adc17 protein levels from the WT, namely: Figure 1E, 6B, 7D, and S4E.

© 2024. Published by The Company of Biologists under the terms of the Creative Commons Attribution License (https://creativecommons.org/licenses/by/4.0/).

3. “Three of the mutants, wsc1Δ, wsc3Δ, and wsc4Δ showed a mild to moderate growth defect. The most severely affected strain, wsc1Δ, has previously been implicated in Mpk1 activation following rapamycin treatment (Krause and Gray, 2002). We did not observe an Mpk1 activation defect using our wsc1Δ strain and conditions however (Figure 1C).” it seems to me mtl1 also has mild phenotype. wsc1 has strong phenotype since I cannot see any growth on the rapamycin treatment.

We have increased the brightness on this figure so that the residual *wsc1Δ* growth can be seen more clearly. The *mtl1Δ* strain does not have a consistent growth defect across samples, and growth of this strain is slightly better than either the *wsc3Δ* or *wsc4Δ* mutants within the image shown.

4. “Reasoning that there may be functional redundancy between the Wsc1, Wsc3, and Wsc4 sensor proteins, we made a wsc1/3/4Δ strain. We found that wsc1/3/4Δ cells are incapable of growth in the presence of rapamycin (Figure 1D) and have greatly diminished Mpk1 activation following rapamycin treatment (Figure 1E).” While there is perhaps some redundancy to Mpk1 signalling it is not growth assay. Maybe make it clear in 1B that is some growth left?

We have increased the brightness of the images in Figure 1B to clarify that there is still some growth with the *wsc1Δ* strain.

5. Combining these two features was insufficient to achieve Adc17 expression in the absence of rapamycin (Figure 3B). please label the lanes and refer to them when describing the data. Also describe the data in figure 3B a bit more elaborate. E.g. there is no mention on P-Rps6 control.

We have labelled the lanes and added further explanation for this figure, which is now Figure 5B, from line 198:

“Cells containing empty vector alone had no change in Adc17, P-Mpk1, P-Rps6 (TORC1 activation) or Mkk1-DD protein expression after 4 hours of galactose supplementation (compare lanes 1 and 2), while rapamycin treatment had the expected effect (lane 3). Galactose supplementation therefore does not activate the CWI pathway and proteasome assembly chaperone production. Cells containing the vector saw increased levels of Mkk1-DD and, as expected, P-Mpk1 (compare lanes 4 and 5), to an even greater extent than when they were treated with rapamycin (lane 6). The effects on TORC1 activity (P-Rps6) were far less dramatic however, and Adc17 levels were not increased, unlike when cells were treated with rapamycin (compare lanes 5 and 6).”

6. Excitingly, there was a notable decrease in Adc17 expression in Sch9-2D3E expressing cells, suggesting a role for Sch9 inhibition (Figure 3C). Please define the Sch9 mutant plus reference. I assume this is a hyperactive mutant. What happens in Sch9 delete ?

We have defined the mutant within the text, including a reference, as suggested on line 155:

“…a constitutively-active phospho-mimetic Sch9-2D3E (Sch9$^{T723D;S726D;T737E;S758E;S765E}$) construct (Urban et al., 2007)”

We have added further references to this being a constitutively active form of Sch9 throughout the text.

Based on the reviewers suggestion, we assessed what happened in the case of the absence of Sch9, intriguingly finding that this too led to a decrease in Adc17 induction following rapamycin treatment (Figure S4D), noting this at line 220:

“Notably, *sch9Δ* cells also had a defect in Adc17 production, but not Mpk1 activation (Figure S4D-

© 2024. Published by The Company of Biologists under the terms of the Creative Commons Attribution License (https://creativecommons.org/licenses/by/4.0/).

E), demonstrating the importance of Sch9 activity modulation for proteasome assembly chaperone production."

7. There are notable questions that merit discussion and potential experimental investigation. For instance, what are the effects in the sch9D mutant? Additionally, how do wcs/1/3/4 + Sch9 hyperactive mutants respond?

As noted above we have now investigated the effect of sch9 knockout. We have also expressed the sch9-2D3E construct in the wsc1/3/4 mutant to assess if this results in even lower Adc17 production (Figure 6F), but we did not observe an additive effect, as noted and discussed at line 232:

"Maintaining Sch9 activation had no effect on Mpk1 activation in the *wsc1/3/4Δ* cells, or Adc17 protein induction. CWI pathway activation through Wsc1, Wsc3, and Wsc4 is therefore the major regulatory pathway in *ADC17* mRNA translation, while Sch9 inhibition has an important role in modulating the response"

8. If I understand correctly, TORC also regulate Mpk1. If so, it seems important to depict this relationship in Figure 4E.

This relationship is depicted indirectly through TORC1's regulation of the CWI pathway through Wsc1, Wsc3, and Wsc4. We do not show a more direct pathway as our data suggests this is the major way the regulation of Mpk1 by TORC1 works.

*****
Reviewer 3 Advance Summary and Potential Significance to Field:
The work of Williams et al. reports on studies aimed at defining the relations between TORC1 and CWI signalling and its effect on proteasome function. The authors report on a considerable amount of work with experiments from different viewpoints and used a variety of mutants and expression constructs of different genes. However, in contrast to what is claimed in the abstract and in results and discussion, a clear picture of what the interactions are and what they mean cannot be derived. This is partially due to some basic errors and lack of proper controls, as listed in the following:

Reviewer 3 Comments for the Author:
1.A major drawback of this study is the perception, that Wsc4 would be a cell wall sensor residing in the plasma membrane (depicted in Fig. 2G). In fact, this protein localizes at the ER and has different functions from the sensors (see for example SGD database or any of the reviews cited). Thus, there really are only 5 (and not 6) CWI sensors, as claimed in line 83.

The initial study describing the localisation of Wsc4 to the ER (https://doi.org/10.1074/jbc.274.16.11296) relies on a very short-term induction of a tagged version of the protein under gal-inducible expression. As far as we are aware this localisation study has not been repeated. We do not believe this is the best way to look at localisation of an endogenous protein, and so have repeated this experiment using endogenously tagged Wsc4- GFP, finding it at the cell cortex (Figure S1A). We therefore treat this as a bona-fide CWI sensor protein in this study, and have discussed our reasoning at line 85:

"We included Wsc4 in this analysis as although previous work using short-term induction assigned its localisation to the ER, an endogenous knock-in we made had a cortical localisation (Figure S1A)."

[2.In]2.In this context, and beyond, reasoning from line 90ff must be considered. First, it is not clear why the authors choose wsc1 wsc3 wsc4 triple mutants to study redundancy. From previous works it would appear that Wsc1, Wsc2, and Wsc3, in that very order, exert the important functions. It would thus have been logic to use at least the triple deletion of those. As Wsc4 has

© 2024. Published by The Company of Biologists under the terms of the Creative Commons Attribution License (https://creativecommons.org/licenses/by/4.0/). 

probably no function in what the authors are studying (see above), a minimum to test would have been the double deletion wsc1 wsc3. Again, I would strongly suggest to also test wsc1 wsc2.

Our evidence shows that Wsc4 has a cortical localisation and thus should be included in this analysis. We have now included analysis of double mutants of Wsc1 with each of these three deletions in supplementary figure 1. Deletion of Wsc2 did not enhance the observed growth defect in *wsc1Δ* cells, while deletion of either Wsc3 or Wsc4 did. However, these double deletions showed no differences in Mpk1 activation or Adc17 production. Only the triple mutant showed these defects, and so we conclude that all three of these proteins are redundantly promoting activation of the CWI pathway in response to rapamycin treatment.

With this in mind, the statement “These data suggest that TORC1 inhibition activates the CWI through the Wsc1, Wsc3, and Wsc4 sensor proteins.” is clearly an unwarranted overinterpretation.

We believe we have solidified this point through the above-described experiments, and hope the reviewer now agrees with us.

[3.In]3.In line 103ff the authors test the hyperactivation of Mpk1 by a mutant allele of MKK1 expressed under an inducible GAL promoter. This means switching carbon sources, which is a problem by itself in this context. What is missing in this experiment is a control of the wild-type MKK1 allele expressed under the same promoter, which would be expected to have no effect.

This experiment was designed to assess whether activation of Mpk1 causes the actin depolarisation effect seen after treatment with rapamycin by decoupling the inhibition of TORC1 from the activation of Mpk1. Low levels of Mpk1 activity (as in the basal state) are associated with high levels of cellular actin polarisation. If Mpk1 activation did induce depolarisation, we would expect there to be an effect of actin depolarisation in cells with galactose-induced expression of Mkk1-DD. If we had seen such an effect, the reviewer is correct that the switching of Carbon source could have been responsible. However, as we observed no change (i.e. a negative result) in actin polarisation with the Mkk1-DD allele following the change of carbon source (Figure S2D-E) we do not expect that a WT version of Mkk1 would be any different and view the control as unnecessary. Of course, if we had observed a difference we agree that the suggested control would have been required.

4. Line 109 states “CWI-Bni1-actin polarity section deleted as suggested, ready for the reviewers if required.” – What is this? – Suggested by whom?

Line 109 has been deleted. The data it referred has been deleted from the paper to remove complexity and improve the flow of the story.

5. Line 132ff reports on Zymolyase sensitivity. The conclusion is an effect on the cell wall. To really conclude that (line 150ff), both cell wall carbohydrates and cell wall protein concentrations need to be compared between the strains.

The reviewer is correct that we cannot say with certainty what has changed. We have edited the text to reflect that we don’t know what changes are occurring, but that they are likely at the cell wall, as this is what is targeted by the Zymolyase enzyme cocktail at line 144:

“This indicates that there is no requirement for CWI activation for the changes responsible for increased zymolyase resistance. This is likely occurring through changes to the structure or composition of the cell wall.”

6. Line 157ff: To induce CWI signalling, calcofluor white is employed as a “well described activator”. So this is true, it is also affecting cell wall signalling in a rather indirect way in interacting with chitin. The authors may want to consider repeating these experiments with a

© 2024. Published by The Company of Biologists under the terms of the Creative Commons Attribution License (https://creativecommons.org/licenses/by/4.0/).

direct glucanase inhibitor of the echinocandin class.



We thank the reviewer for this suggestion. We have used the echinocandin micafungin for this, finding similar results to calcofluor white treatment, from line 180:

"Activating the CWI pathway using micafungin, an echinocandin which inhibits ß-1,3-glucan production, similarly induced Mpk1 activation and Adc17 protein production (Figure S4A)."

7. Line 168: Tethering of the mRNA to actin should be explained in more detail. How is this done? - Is there proof that tethering does not interfere with translation efficiency?

The method used has been explain further in the text:

"We tethered *ADC17* mRNA containing PP7 stem loops in the 3'UTR to actin patches by expressing the PP7 binding protein fused to GFP in cells containing a nanobody against GFP fused to the CAP protein Abp1 (Figure 5A, top), which we have previously shown can support a high level of Adc17 protein production (Williams et al., 2022)."

The tethered mRNAs are able to be translated efficiently following rapamycin stress, as can be seen in lanes 3 and 6 of Figure 5A.

8. Interaction of Mpk1 activation with TORC1/Sch9 signalling: I think the small GTPase Rho5 was shown to work as a negative regulator of CWI signalling and also shows synthetic lethality with sch9 deletions? - The authors may want to consider this as the unknown "additional factor"? - At least it merits discussion.

We thank the reviewer for drawing our attention to Rho5. We have discussed the possible role of Sch9 further, including by reanalysing some data on the number of visible mRNA punctae in the Sch9-2D3E expressing cells for Figure S4F at line 226:

"The number of mRNA punctae was similar between the WT and Sch9-2D3E, indicating that the decrease in Adc17 protein production is unlikely due to a change in *ADC17* mRNA abundance (Figure S5F)."

We have further dedicated a section of the discussion to this topic, from line 294:

"S6Kinases like Sch9 have been shown to promote general translation through promoting Ribosome Biogenesis and promoting both ribosome scanning to the initiation codon and elongation (Williams and Rousseau, 2024). It is possible that by altering these translational processes Sch9 inhibition can change the types of mRNAs being translated. Sch9 has also been genetically linked with Rho5, with a double deletion being synthetically lethal, a phenotype suggested to occur through the subsequent uncontrolled activation of the common target Rim15. Rho5 is a small GTPase which becomes GTP bound upon stress and acts to limit CWI activation (Novarina et al., 2021; Schmitz et al., 2002; Schmitz et al., 2015). It is possible that constitutive Sch9 activation interferes with GTP binding of Rho5, either affecting Rho5's role in restricting CWI activation or another role important for Adc17 translation. The exact role of Sch9 will be explored in future work."

[9.The]9.The authors speak frequently of ADC17 expression. Expression is a term that should be reserved to transcription of genes, but is often confused with the amount/production of proteins by biochemists in the literature. Especially in this manuscript, this practice is missleading, as it becomes clear only by careful reading of the text, that what is meant is the translation of pre-formed mRNA, rather than transcription. Phrasing should thus make clear if they talk about gene expression, translation efficiency, or protein production/concnetration.

Our use of the word expression throughout the text was as a general term to cover everything leading to the production of protein. We accept that this could be confusing and have therefore

© 2024. Published by The Company of Biologists under the terms of the Creative Commons Attribution License (https://creativecommons.org/licenses/by/4.0/).

changed “expression” to “protein expression”, “translation”, “levels”, or “production” throughout the text for clarity.

10.Methods: line 322 “Cells were grown overnight ...” – Is there any way the authors made sure that cells are still growing logarithmically and were in the same growth phase for comparisons? – At least OD600 should be determined.

We indeed made sure our cells were in log phase for all experiments. We have made the text clearer on this point at line 460:

“For all experiments cells were grown overnight prior to being diluted to 0.2 OD600 nm, grown to 0.4-0.6 OD600 nm and used as described below.”

line 342ff: surely cells were not washed with 600/400 ml of buffer etc. Please correct for microliters

This has been corrected.

line 348: likewise, 30 mg of protein would be hard to lead on a gel. I suspect that 30 microgramms of total protein are meant?

The reviewer is correct. This has been corrected.

line 364ff: is this an accepted method to determine Zymolyase sensitivity? – If so, please give a reference.

The zymolyase protocol is adapted from a previous protocol, which we have now referenced in line 513:

“We adapted and simplified a previously described protocol for our assay (Kuranda et al., 2006).“

We thought the reviewer would appreciate some more detail on the experimental protocol, so have expanded it in the following lines:

“1 OD600 nm of log phase cells, either treated with rapamycin for the indicated time or not, were spun down at 7200g for 3 minutes, and washed twice in 5 ml water. Cells were then resuspended in 1 ml 0.1M Tris (pH7.4), and the OD600 nm measured before and after 45 minutes incubation, unless otherwise indicated, at 37$^{O}$C with 1.3 Units Zymolyase (Stratech, MP Bioscience).”

We have further included our initial experiments which identified 45 minutes as the timepoint to use in Figure 3A.

In summary, while the authors performed an admirable amount of work, for this reviewer the actual progress in the field does not become evident. Where there are clear conclusions drawn into this direction, they do not seem to be warrented by the experimental data.

****

We hope the clarifications we have added have addressed the reviewers concerns and that they now support our findings. We look forward to hearing back from you.

Thank you,

Thomas Williams

© 2024. Published by The Company of Biologists under the terms of the Creative Commons Attribution License (https://creativecommons.org/licenses/by/4.0/).

Second decision letter

MS ID#: JOCES/2023/261892

MS TITLE: Distinct TORC1 signalling branches regulate Adc17 proteasome assembly chaperone expression

AUTHORS: Thomas David Williams, Ifeoluwapo Joshua, Flavie Soubigou, Sylwia Dublanska, Rebecka Bergquist, and Adrien Rousseau
ARTICLE TYPE: Research Article

I am happy to tell you that your manuscript has been accepted for publication in Journal of Cell Science, pending standard publication integrity checks.

Reviewer 1

*Advance summary and potential significance to field*

This manuscript entitled “Distinct TORC1 signaling branches regulate Adc17 1 proteasome assembly chaperone expression.” By Thomas D. Williams et al. describes the signaling pathway involved in Adc17 translation controlling during TROC1 inhibition. They showed that the activation of the Cell Wall Integrity pathway and sch9 inhibition are both required for this phenomenon.

*Comments for the author*

The authors properly responded to the comments.

Reviewer 2

*Advance summary and potential significance to field*

The authors demonstrate that TORC1 inhibition alters cell wall properties inducing changes by activating the Cell Wall Integrity pathway through the Wsc1 Wsc3, and Wsc4 sensor proteins. They show that these signals alone are insufficient to drive protein expression. Additionally, they identify that inhibition of Sch9 is also required. The work broadens our understanding of the signaling pathways that regulate proteasome assembly chaperone production.

*Comments for the author*

The reviewers addressed most of my comments. However, I still believe that using a loading control other than Ponceau staining would be better since Ponceau staining is not truly quantitative. Nevertheless, I appreciate the effort put into providing the quantifications. I only noted a minor issue: the yeast nomenclature for genotypes, mRNA (Italic and capitals) and protein names ((not italic, first letter capital rest lowercase) is not always consistent throughout the manuscript.
It would be good to check this once more.

Reviewer 3

*Advance summary and potential significance to field*

The summary from my first review is still valid, as very few additions have been made to the revised version, which do not change the potential significance of the manuscript to the field.

*Comments for the author*

© 2024. Published by The Company of Biologists under the terms of the Creative Commons Attribution License (https://creativecommons.org/licenses/by/4.0/).

The authors have answered all my queries to the first version of the manuscript, adapted the text as suggested, and have added additional experiments to my full satisfaction. Thus, I have no further comments.

© 2024. Published by The Company of Biologists under the terms of the Creative Commons Attribution License (https://creativecommons.org/licenses/by/4.0/).
